# Nano-Assemblies from Amphiphilic PnBA-b-POEGA Copolymers as Drug Nanocarriers

**DOI:** 10.3390/polym13071164

**Published:** 2021-04-05

**Authors:** Angeliki Chroni, Thomas Mavromoustakos, Stergios Pispas

**Affiliations:** 1 Theoretical and Physical Chemistry Institute, National Hellenic Research Foundation, 48 Vassileos Constantinou Avenue, 11635 Athens, Greece; angelikechrone@gmail.com; 2 Department of Chemistry, National and Kapodistrian University of Athens, Panepistimioupolis, 15771 Zografou, Greece; tmavrom@chem.uoa.gr

**Keywords:** amphiphilic block copolymers, polymeric nanocarriers, drug delivery systems, thin film hydration method, drug encapsulation, drug release profile, drug–polymer intermolecular interactions

## Abstract

The focus of this study is the development of highly stable losartan potassium (LSR) polymeric nanocarriers. Two novel amphiphilic poly(n-butyl acrylate)-block-poly(oligo(ethylene glycol) methyl ether acrylate) (PnBA-b-POEGA) copolymers with different molecular weight (M_w_) of PnBA are synthesized via reversible addition fragmentation chain transfer (RAFT) polymerization, followed by the encapsulation of LSR into both PnBA-b-POEGA micelles. Based on dynamic light scattering (DLS), the PnBA_30_-b-POEGA_70_ and PnBA_27_-b-POEGA_73_ (where the subscripts denote wt.% composition of the components) copolymers formed micelles of 10 nm and 24 nm in water. The LSR-loaded PnBA-b-POEGA nanocarriers presented increased size and greater mass nanostructures compared to empty micelles, implying the successful loading of LSR into the inner hydrophobic domains. A thorough NMR (nuclear magnetic resonance) characterization of the LSR-loaded PnBA-b-POEGA nanocarriers was conducted. Strong intermolecular interactions between the biphenyl ring and the butyl chain of LSR with the methylene signals of PnBA were evidenced by 2D-NOESY experiments. The highest hydrophobicity of the PnBA_27_-b-POEGA_73_ micelles contributed to an efficient encapsulation of LSR into the micelles exhibiting a greater value of %EE compared to PnBA_30_-b-POEGA_70_ + 50% LSR nanocarriers. Ultrasound release profiles of LSR signified that a great amount of the encapsulated LSR is strongly attached to both PnBA_30_-b-POEGA_70_ and PnBA_27_-b-POEGA_73_ micelles.

## 1. Introduction

Nano-drug delivery systems (NDDSs) have revolutionized drug delivery in the last few decades, endowing drugs with increased stability and water solubility, prolonging the cycle time, enhancing the uptake rate of target cells or tissues, reducing enzyme degradation, and controlling the release of the drug [1,2,3,4].

The NDDSs used in modern biomedicine concern supramolecular self-assembled structures divided into organic, inorganic, and composite materials. Amphiphilic block copolymers (AmBCs) have been the workhorse of pharmaceutical nanotechnology in recent years towards the design of drug-loaded block copolymer nanocarriers for the demanding treatment of crucial diseases [5,6,7,8]. The delivery of drugs, proteins, and nucleotides is possible via AmBCs self-assembled into biocompatible nanostructured multifunctional structures such as micelles, polymer containing liposomes, or polymeric nanoparticles [9].

A multitude of applications in nanomedicine and diagnostics are powered by AmBCs [10,11,12]. The unique core-shell architecture of AmBC nanoassemblies enables a physical encapsulation of drug molecules into their hydrophobic micellar core, conferring an enhanced drug solubility and reduced toxicity. The hydrophilic shell stabilizes the core, ameliorates drug pharmacokinetics, and facilitates drug accumulation in tumors through the enhanced permeability and retention (EPR) effect [13,14].

Poly[oligo(ethylene glycol) methyl ether methacrylate)] (POEGMA) and poly[oligo(ethylene glycol) methyl ether acrylate)] (POEGA) are hydrophilic biocompatible polymers due to their oligo(ethylene glycol) (OEG) side groups. To date, several scientific works have utilized POEG[M]A blocks for the formation of micelles from AmBCs [15,16,17]. Moreover, the OEG fragment endows polymeric systems with “stealth” properties, preventing their recognition by the immune system [18,19].

The stability of AmBC micelles in serum media is vital to their use in drug delivery, where dilution after intravenous injection usually elicits micellar disassembly and drug unloading [20,21]. To the best of our knowledge, few studies are focused on investigating the colloidal and physicochemical stability of polymeric micelles in biological fluids. To date, highly skilled, often time-consuming, and expensive techniques are usually applied for the elucidation of protein–polymer interactions, including surface plasmon resonance, in situ ellipsometry, total internal reflection fluorescence spectroscopy, ATR-FTIR (attenuated total reflection Fourier transform), etc. [22,23,24]. Hence, a simple, quick, and cost-effective method is needed for the investigation of the stability of polymeric micelles in a biological environment by detecting potential alterations of their physicochemical characteristics in serum proteins.

A well-known industrial and environmental technique that places great promise in the fields of materials science and medicine for producing complex polymers and polymeric nanoparticles with controlled molecular weight (M_w_) and narrow molecular weight distributions (M_w_/M_n_) is reversible addition fragmentation chain transfer polymerization (RAFT) [25].

Block copolymer nano-assemblies obtained via RAFT enable a physical encapsulation of hydrophobic therapeutics into their hydrophobic micellar core. Sartans medicines have been developed in parallel as additional therapeutics for the rennin–angiotensin system (RAS), including new antihypertensive drugs (losartan, valsartan, candesartan, irbesartan, olmesartan, telmisartan, eprosartan, azilsartan), which compete with the hypertensive action of the vasoconstrictor hormone angiotensin II (ANG II) at the ANG II type 1 (AT1) receptor site, modulating the RAS [26,27,28,29,30]. Losartan potassium (LSR) is an ANG II receptor blocker whose major capability relies on hypertension treatment [31,32]. Yet, few studies have shed any light on the association of LSR with malignancy by reporting that the encapsulation of LSR in peptide hydrogels and liposomes ameliorates the efficacy of chemotherapy and highlighting its use in reducing breast cancer recurrence [33,34,35]. AT1 antagonists, however, meet formidable challenges regarding their lipophilicity. Block copolymer nanocarriers come to the rescue, increasing the bioavailability of AT1 antagonists.

Hitherto, the examination of micelle structure and polymer–drug intermolecular interactions using nuclear magnetic resonance (NMR) spectroscopy is scarce in existing research for rational polymeric nanocarrier design and drug encapsulation [26,36]. According to our previous work, NMR spectroscopy proved a robust tool for the structural elucidation of the micelles and the detection of the intra- and intermolecular interactions between the copolymer and the drug [37].

Here, two novel biocompatible AmBCs with different M_w_ of PnBA, namely poly(n-butyl acrylate)-block-poly(oligo(ethylene glycol) methyl ether acrylate) (PnBA-b-POEGA), are synthesized using RAFT polymerization. Νovel drug delivery nanocarriers are subsequently developed by encapsulating LSR into PnBA_30_-b-POEGA_70_ and PnBA_27_-b-POEGA_73_ (where the subscripts denote wt.% composition of the components) diblock copolymers. The assessment of critical micelle concentration (CMC), size, shape, surface potential, and structure of the PnBA-b-POEGA diblocks and LSR-loaded PnBA-b-POEGA nanocarriers were determined using a variety of techniques. The LSR-loaded PnBA-b-POEGA nanocarriers (20 wt.% and 50 wt.% concentration of LSR in the mixture) were prepared using the thin film hydration method (TFHM), and they are referred in the text as PnBA_30_-b-POEGA_70_ + 20% LSR, PnBA_30_-b-POEGA_70_ + 50% LSR and PnBA_27_-b-POEGA_73_ + 20% LSR, PnBA_27_-b-POEGA_73_ + 50% LSR. The stability in fetal bovine serum (FBS) of PnBA_30_-b-POEGA_70_ and PnBA_27_-b-POEGA_73_ micelles was examined by dynamic light scattering (DLS) to gain further insight into the protein–polymer interactions. A plethora of NMR experiments were performed to explore the intramolecular interactions between the PnBA_30_-b-POEGA_70_ micelles and LSR, and to discover the self-diffusion coefficients D of the PnBA_30_-b-POEGA_70_ + 50% LSR nanocarriers. Moreover, temperature studies using ^1^H-NMR spectroscopy for the POEGA hydrophilic homopolymer and the PnBA-b-POEGA micelles over the temperature range 25–80 °C were further conducted to trace the mobility of protons located in the hydrophobic PnBA micellar core. At last, drug loading (DL%) and encapsulation efficiency (%EE) were calculated by means of ultraviolet visible (UV-Vis) spectroscopy, followed by the ultrasound-triggered release of LSR from PnBA_30_-b-POEGA_70_ and PnBA_27_-b-POEGA_73_ micelles.

## 2. Materials and Methods

### 2.1. Materials

The following materials were used in our study: n-butyl acrylate, (nBA, 99%, Sigma-Alrdrich, Athens, Greece), oligo(ethylene glycol) methyl ether acrylate (OEGA, 98%, Sigma-Alrdrich, Athens, Greece) with average Μ_w_ = 480 g/mol, 2,2-Azobis(isobutyronitrile) (AIBN, 98% Sigma-Alrdrich, Athens, Greece), 2-(dodecylthiocarbonothioylthio)-2-methylpropionic acid (DDMAT, 98% Sigma-Alrdrich, Athens, Greece), pyrene (Py, 98%, Sigma-Alrdrich, Athens, Greece), fetal bovine serum (FBS, Sigma-Alrdrich, Athens, Greece), losartan potassium (LSR, 99.8%, Rafarm, Athens, Greece), 1,4-dioxane (99.8%, Sigma-Alrdrich, Athens, Greece), n-hexane (Hx, 97%, Sigma-Alrdrich, Athens, Greece), tetrahydrofuran (THF, 99.9%, Sigma-Alrdrich, Athens, Greece), acetone (ACTN, 99.5%, Sigma-Alrdrich, Athens, Greece), methanol (MeOH, 99.9%, Sigma-Alrdrich, Athens, Greece), phosphate-buffered saline tablets (PBS, 98%, Sigma-Alrdrich, Athens, Greece), water for injection (WFI, 99%, Sigma-Alrdrich, Athens, Greece), and deuterium oxide (D_2_O, 99%, Sigma-Alrdrich, Athens, Greece).

### 2.2. Synthesis of PnBA-b-POEGA Diblock Copolymers

The hydrophobic PnBA homopolymers were synthesized in 4000 and 7800 g/mol M_w_ variations in a previous work, and used as macro-CTAs for commencing the subsequent polymerization of the OEGA monomer [38].

The synthesis of PnBA_30_-b-POEGA_70_ and PnBA_27_-b-POEGA_73_ block copolymers involved AIBN as the initiator, PnBA (4000 and 7800 g/mol) homopolymers as the macro-CTAs, and 1,4-dioxane as the solvent. A typical RAFT polymerization procedure for the synthesis of the PnBA_30_-b-POEGA_70_ diblock in 4000 molar mass of PnBA is described as follows: to a round-bottom flask, 25 mL, PnBA (0.38 g), AIBN (0.00314 g, 0.019 mmol), OEGA (480 g/mol, 0.89 g), and 6.4 mL of 1,4-dioxane were added with a magnetic stirrer and fitted with a septum. The final solution was degassed by nitrogen gas bubbling for 15 min and placed in a thermostatted oil bath at 70 °C for 24 h. After the polymerization reaction, the product was precipitated in 10-fold excess of n-hexane and dried in a vacuum oven for 48 h (yield: 65%). The dry PnBA_30_-b-POEGA_70_ diblock was collected and stored.

### 2.3. Preparation of Self-Assembled PnBA-b-POEGA Diblock Copolymers

Despite the fact that PnBA_30_-b-POEGA_70_ and PnBA_27_-b-POEGA_73_ block copolymers are dissolved directly in aqueous media, their micelles were prepared using the thin film hydration method (TFHM). The contradiction is explained as LSR-loaded nanocarriers were also prepared with TFHM (more details are given in Section 2.5). Specifically, 10 mg of each diblock was dissolved in ACTN (stock solution) and allowed to stand for polymer dissolution. Afterwards, each polymer stock solution was transferred into a flask and placed in a rotary evaporator for the efficient evaporation of ACTN until a thin film of each polymer was formed around the inner part of the flask. When the thin film was formed, 10 mL filtered water for injection (WFI) was added to the flask and stirred until the entire thin film was dissolved. The concentration of the aqueous stock solutions was 10^−3^ g/mol.

### 2.4. FBS Interactions with PnBA-b-POEGA Diblock Copolymers

The mixtures of PnBA_30_-b-POEGA_70_ and PnBA_27_-b-POEGA_73_ copolymers with clarified FBS were prepared in filtered PBS using two protocols based on different polymer dilution to FBS and different FBS:PBS ratios. The copolymer stock solutions were prepared with direct dissolution in filtered WFI at a concentration of 3 × 10^−3^ g/mL for both protocols. According to the first protocol, 50 μL of each diblock was mixed with 3 mL FBS:PBS (1/9 *v*/*v*) ratio. The second protocol included the mixing of 100 μL sample with (a) 3 mL FBS:PBS (1/9 *v*/*v*) ratio and (b) 3 mL FBS:PBS (1/1 *v*/*v*). All FBS-copolymer mixtures were filtered through 0.45 μm pore size filters and allowed to stand 1 h for equilibration before DLS measurements.

### 2.5. Preparation of LSR-Loaded PnBA-b-POEGA Nanocarriers

**Scheme 1:**
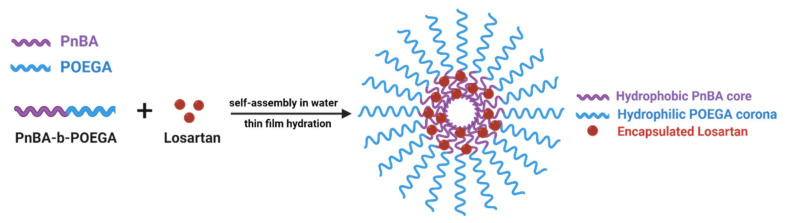
Schematic illustration for the preparation of LSR-loaded PnBA-b-POEGA nanocarriers using the TFHM in aqueous solutions.

The LSR-loaded PnBA_30_-b-POEGA_70_ + 20% LSR, PnBA_30_-b-POEGA_70_ + 50% LSR and PnBA_27_-b-POEGA_73_ + 20% LSR, PnBA_27_-b-POEGA_73_ + 50% LSR nanocarriers were prepared using the TFHM. Specifically, an appropriate amount of LSR was dissolved in ACTN to prepare 20 wt.% and 50 wt.% concentrations of LSR in each copolymer–drug mixture. Afterwards, each copolymer and LSR stock solution was mixed in the appropriate amounts. Each copolymer–drug mixture was transferred into a flask and placed in a rotary evaporator for the efficient evaporation of ACTN until a thin film of each copolymer and LSR was formed around the inner walls of the flask. Then, 5 mL filtered WFI was added to the flask and stirred until the entire thin film was dissolved. The concentrations of the aqueous stock solutions were 10^−3^ g/mol. All copolymer and drug-loaded solutions were filtered through 0.45 μm pore size filters and allowed to stand overnight for equilibration before measurements. A schematic illustration for the preparation of LSR-loaded PnBA-b-POEGA nanocarriers using the TFHM is proposed in Scheme 1.

### 2.6. Drug Loading and Encapsulation Efficiency Calculations of LSR

The percentage of LSR incorporated into PnBA_30_-b-POEGA_70_ + 50% LSR and PnBA_27_-b-POEGA_73_ + 50% LSR nanocarriers was estimated by UV-Vis (Perkin-Elmer, Lambda 19 spectrophotometer, Waltham, MA, USA) spectroscopy. LSR concentration was calculated with the aid of the following LSR calibration curve in ACTN, which was on the general form y = ax + b (where a is the slope and b is the intercept):

LSR concentration (g/mL) = (y − 0.22504)/98,462.16599(1)

The absorbance (y) of the drug–polymer solutions was measured at 205 nm. %DL is the amount of drug loaded per unit weight of the micelle/nanoparticle, and is calculated by the amount of total entrapped drug divided by the total micelle/nanoparticle weight. %EE is the percentage of drug that is successfully entrapped into the micelle/nanoparticle, and is calculated by the total encapsulated drug divided by the total drug added. %DL %EE were calculated using the following Equations:

%DL = [encapsulated drug/total micelles weight (initial)] × 100(2)

%EE = [encapsulated drug/total drug added (initial)] × 100(3)

### 2.7. Ultrasound Release Studies

The release of LSR from PnBA_30_-b-POEGA_70_ and PnBA_27_-b-POEGA_73_ micelles was studied in WFI in an ultrasonic bath sonicator for approximately five hours. Specifically, 5 mL of PnBA_30_-b-POEGA_70_ + 50% LSR and PnBA_27_-b-POEGA_73_ + 50% LSR nanocarriers were added in dialysis bags of 3.5 kDa MWDO. Afterwards, the dialysis bags were inserted into 100 mL filtered WFI and placed in a SOLTEC, SONICA 3300ETH-S3 ultrasonic bath. Aliquots of samples were taken from the external solution at specific time intervals and each time the aqueous solution was restored to its initial volume, so that the reservoir conditions remained constant. On the basis of the calibration curve of the Equation (1), the amount of LSR released at different time intervals, up to 5 h, was determined using UV-Vis (Perkin-Elmer, Lambda 19 spectrophotometer, Waltham, MA, USA) spectroscopy at λ_max_ = 205 nm.

### 2.8. NMR Sample Preparation

For the temperature dependent NMR studies of POEGA homopolymer, PnBA_30_-b-POEGA_70_, and PnBA_27_-b-POEGA_73_ block copolymers, the solutions were prepared as follows: 2 mg of each diblock was directly dissolved in 0.7 mL D_2_O. The PnBA_30_-b-POEGA_70_ micelles and PnBA_30_-b-POEGA_70_ + 50% LSR nanocarriers were prepared by TFHM in D_2_O solutions as follows: 1 mg of PnBA_30_-b-POEGA_70_ diblock was dissolved in 1 mL D_2_O. The partitioning of the drug in the PnBA_30_-b-POEGA_70_ micelles was studied by adding an appropriate amount of LSR (to prepare 50 wt.% concentration of LSR) into the final copolymer–drug mixture.

### 2.9. Evaluation

M_w_ and M_w_/M_n_ of the synthesized PnBA-b-POEGA block copolymers were determined by a size exclusion chromatography (SEC) instrument from Waters Technologies Corporation, Caguas, Puerto Rico. It was equipped with a Waters 1515 isocratic pump, a set of three μ-Styragel mixed bed columns (porosity range between 10^2^ to 10^6^ Å), and a Waters 2414 refractive index detector (equilibrated at 40 °C). Breeze software was utilized for data acquisition and analysis. THF containing 5% *v*/*v* triethylamine was the mobile phase at a flow rate of 1.0 mL/min at 30 °C. The setup was calibrated with linear polystyrene standards, having narrow M_w_/M_n_ and weight average M_w_ in the range of 1200 to 929,000 g/mol. Concentrations in the range 2–4 mg/mL were used for analysis.

NMR studies at different temperatures of PnBA-b-POEGA copolymers were carried out on a Varian 300 MHz NMR spectrometer (Agilent Technologies, Palo Alto, CA, USA) using tetramethylsilane (TMS) as the internal standard and D_2_O as the solvent. The copolymer compositions were also determined by ^1^H-NMR on a Varian 300 MHz spectrometer, using CDCl_3_ as the selective solvent. The composition of PnBA-b-POEGA block copolymers was calculated using the characteristics spectral peaks at 0.92 ppm, which corresponds to the –CH_3_ protons of PnBA side chain (3H, CH_3_), and 3.64 ppm, which corresponds to the –(CH_2_)_9_ protons of POEGA group [36H, (CH_2_-CH_2_)_9_]. ^1^H-NMR spectrum peaks of PnBA_30_-b-POEGA_70_ (300 MHz, CDCl_3_, δ) consist of: 4.17 (g, 2H, CH_2_) POEGA side group, 4.02 (c, 2H, CH_2_) PnBA side chain, 3.64 (h, 2H, CH_2_) POEGA side chain, 3.37 (j, 3H, CH_3_) POEGA side chain, and 2.28 (b, 1H, CH); and 1.89 (a, 2H, CH_2_) PnBA and POEGA main chain, 1.58 (d, 2H, CH_2_) PnBA side chain, 1.37 (e, 2H, CH_2_) PnBA side chain, and 0.92 (f, 3H, CH_3_) PnBA side chain.

^1^H-NMR spectroscopy studies of PnBA-b-POEGA micelles and LSR-loaded PnBA-b-POEGA nanocarriers were performed on a 600 MHz NMR spectrometer (Agilent Technologies, Palo Alto, CA, USA) operated by Vjnmr rev. 3.2A software and with a 5 mm HCN cold probe. Tetramethylsilane (TMS) was the internal standard used, and D_2_O was the solvent. ^1^H NMR spectra were recorded with 65,536 points, 90° pulse, 10 s relaxation delay, and 32 repetitions. Correlated spectroscopy (2D-COSY), Overhauser (2D-NOESY), and diffusion-ordered (2D-DOSY) experiments were reported on a 600 MHz NMR spectrometer. NOESY spectra were recorded at different mixing times with 4096 × 200 points, 1 s relaxation delay, and 32 repetitions per spectrum. The DgcsteSL cc sequence was used to record DOSY spectra with 65,536 points, 1 s relaxation delay, and 16 repetitions. Twenty-four gradient strengths between 0 and 60 gauss/cm were used. All spectra were recorded at 25 °C. Chemical shifts are referenced with respect to the lock frequency and reported relative to TMS.

Fluorescence spectroscopy (FS) experiments were pursued to determine the CMC of the PnBA-b-POEGA block copolymers using a Fluorolog-3 Jobin Yvon-Spex spectrofluorometer, model GL3–21 (HORIBA Scientific, Piscataway, NJ, USA) with pyrene as the fluorescent probe. A stock solution of 1 mM Py in ACTN was prepared and added to the solutions in a ratio of 1 μL per 1 mL polymer solution. The excitation wavelength used for the measurements was 335 nm and emission spectra were recorded in the region of 355–630 nm. Measurements were conducted after the evaporation of ACTN overnight at room temperature.

DLS measurements were performed using an ALV/CGS-3 Compact Goniometer System (ALV GmbH, Siemensstraße 4, 63225 Langen, Hessen, Germany) with an ALV-5000/EPP multi-τ digital correlator with 288 channels and an ALV/LSE-5003 light scattering electronic unit for stepper motor drive and limit switch control. A JDS Uniphase 22-mW He–Ne laser (632.8 nm) was used as the light source. The size data and figures provided in the manuscript are from averaged measurements at 90 degrees, (five measurements per concentration/angle). The obtained correlation functions were analyzed by the cumulants method and CONTIN software (ALVGmbH, Hessen, Germany). All solutions were filtered through 0.45 μm hydrophilic PTFE filters (Millex-LCR from Millipore, Billerica, MA, USA) before measurements. Static light scattering (SLS) measurements were carried out on the same instrument at the same temperature and angular range as in DLS measurements, using toluene as the calibration standard. All samples were prepared at a concentration of 1 × 10^−3^ g/mL. SLS data were determined using Zimm plots.

A ZetaSizer Nanoseries Nano-ZS (Malvern Instruments Ltd., Malvern, UK) was used for the electrophoretic light scattering (ELS) measurements, equipped with a 4 mW solid-state laser at a wavelength of 633 nm and a fixed backscattering angle of 173°. The reported ζ-potential (ζ_pot_) values are the average values of 50 runs, utilizing the Henry approximation of the Smoluchowski equation after equilibration at 25 °C.

UV−Vis absorption spectra of the LSR, LSR-loaded PnBA-b-POEGA nanocarriers, and aqueous solutions of the released LSR were recorded between a 200 and 600 nm wavelength using a Perkin Elmer (Lambda 19) UV–Vis–NIR spectrophotometer (Waltham, MA, USA). The LSR-loaded PnBA-b-POEGA nanocarriers were diluted to get an absorbance value of less than 1. It is notable that the absorption at 254 nm and 228 nm was related to the presence of LSR, and not to the copolymer based on measurements on the pure components.

Mid-infrared (IR) spectra of LSR, PnBA-b-POEGA micelles, and LSR-loaded PnBA-b-POEGA nanocarriers were recorded in the region of 550–4000 cm^−1^ on an Equinox 55 FTIR spectrometer (Bruker, Billerica, MA, USA) equipped with a single reflection diamond ATR accessory (DuraSamp1IR II by SensIR Technologies, Chapel Hill, NC, USA).

Observed ATR-FTIR spectral peaks of PnBA-b-POEGA micelles were: v (cm^−1^) = 2700–3000 (–CH–), 1730 (–C=O–), 1455 (–CH_2_–), 1355 (–CH_3_–), 1107 and 1250 (–C–O–C–), 945, and 850 (–CH_2_–).

## 3. Results

### 3.1. Synthesis and Molecular Characterization of PnBA-b-POEGA Diblock Copolymers

**Scheme 2:**
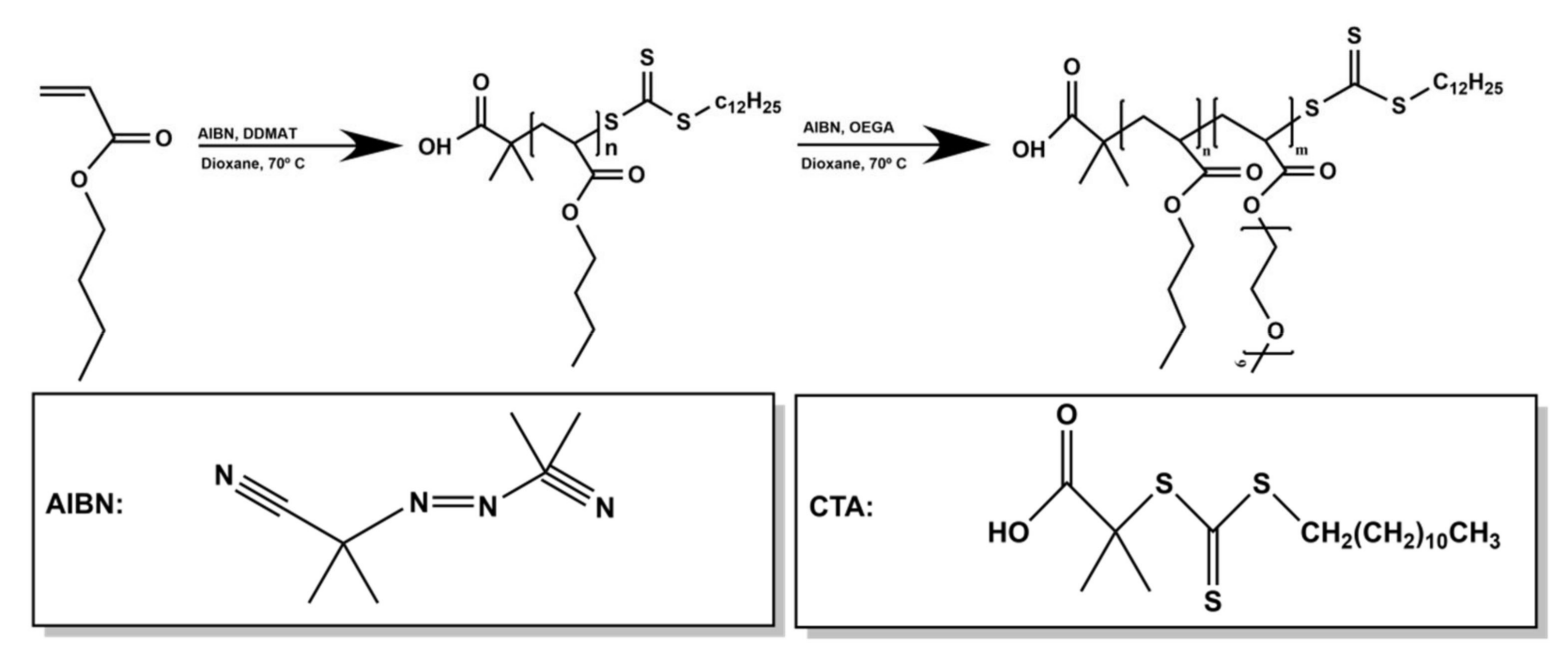
Synthetic route for the preparation of PnBA-b-POEGA diblocks using RAFT polymerization.

**Scheme 3:**
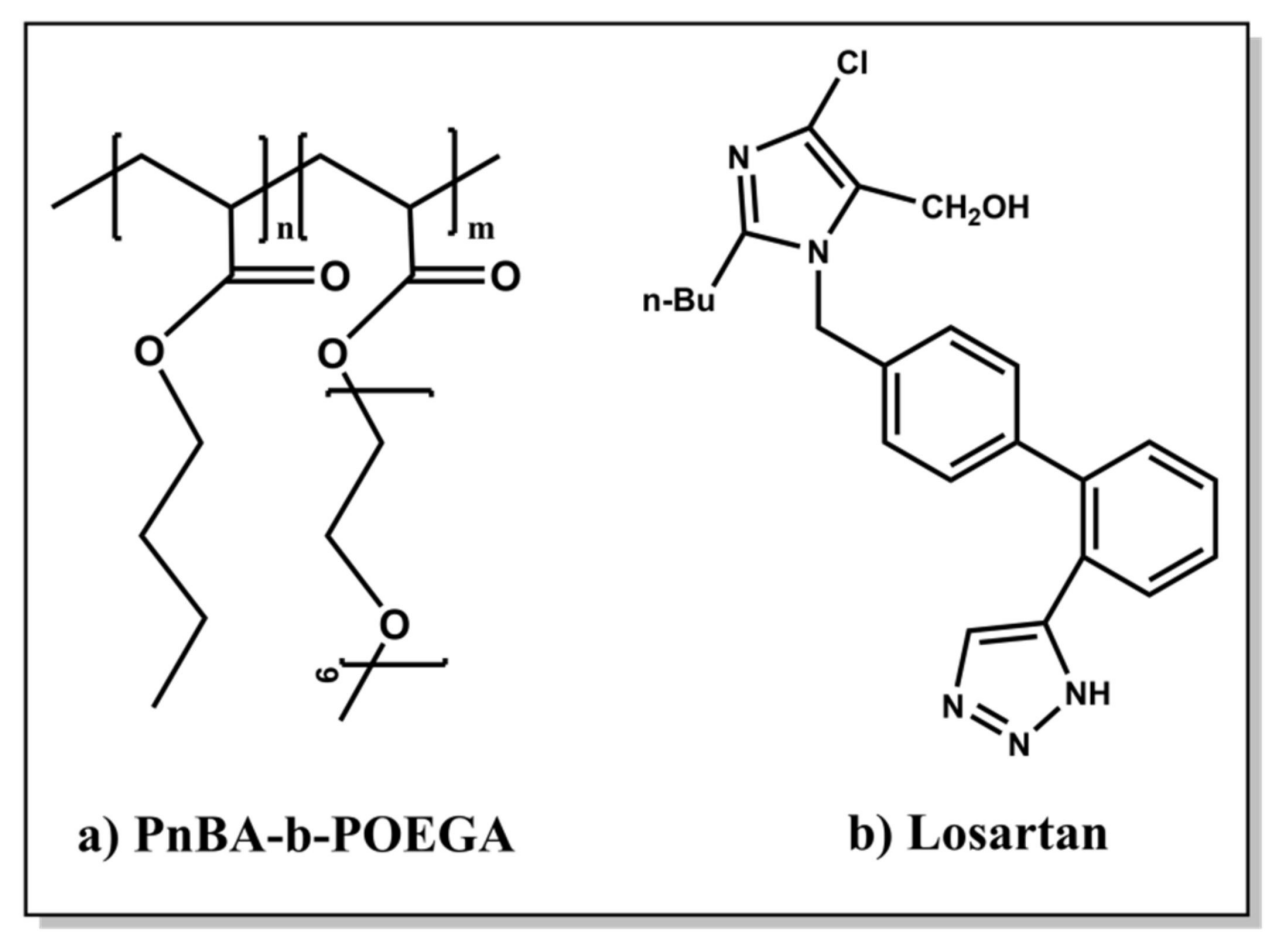
Chemical structures of (**a**) PnBA-b-POEGA diblock copolymers synthesized by RAFT and (**b**) LSR.

**Figure 1:**
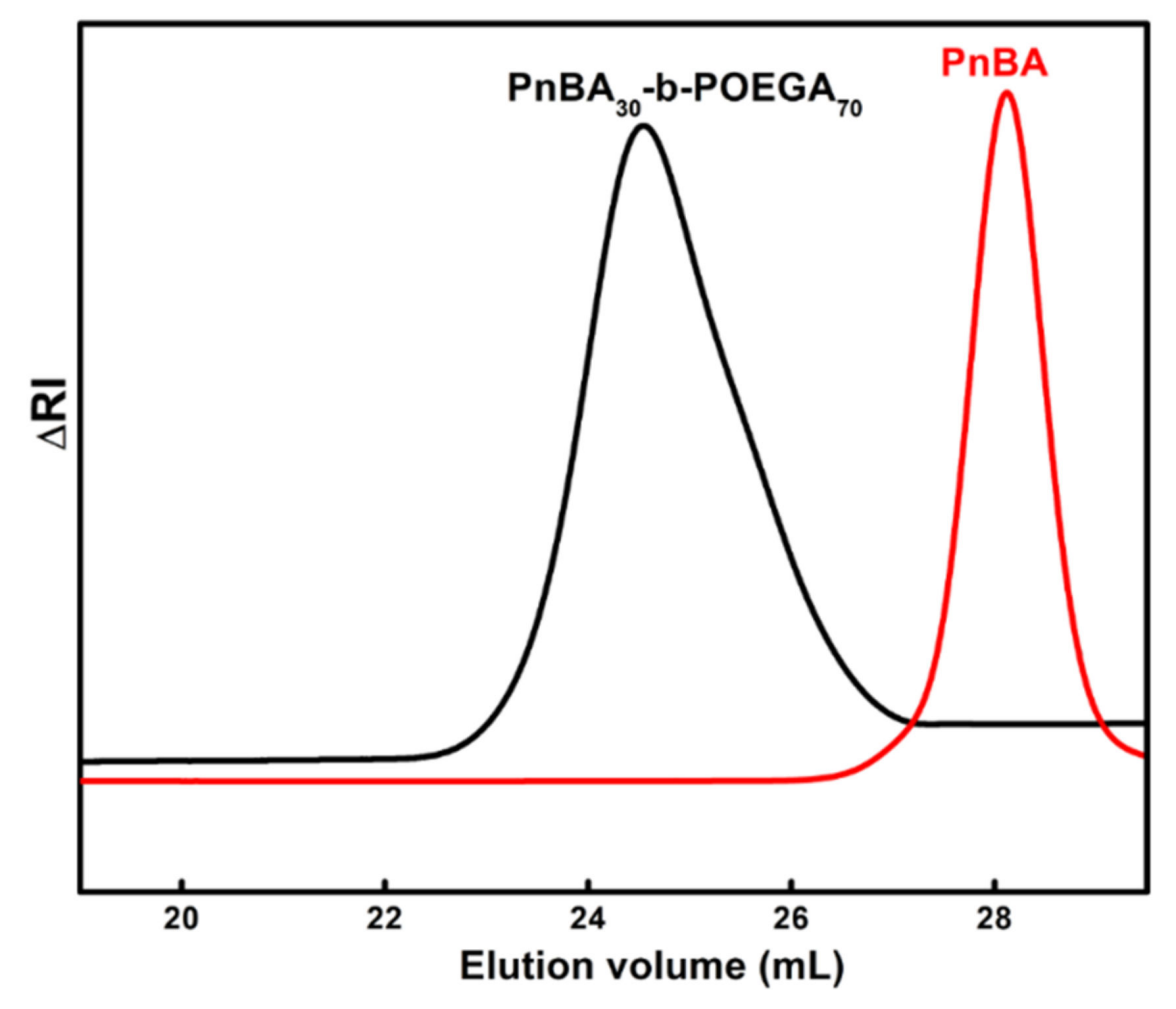
SEC chromatogram depicting the synthesis steps of a PnBA_30_-b-POEGA_70_ diblock.

Two amphiphilic PnBA_30_-b-POEGA_70_ and PnBA_27_-b-POEGA_73_ diblock copolymers were synthesized with different M_w_ of a PnBA block. The PnBA segment enables the formation of equilibrated micellar structures in aqueous media in most cases [39,40,41]. In the current study, the previously synthesized PnBA homopolymers (in 4000 and 7800 g/mol M_w_ variations) [38] were used as macro-CTAs to commence the polymerization of the OEGA. The employment of DDMAT as the CTA leads to polymers with well-controlled M_w_ and M_w_/M_n_ [25,42,43]. The synthetic route of PnBA-b-POEGA copolymers using RAFT polymerization and the chemical structures of the diblocks and LSR are presented in Scheme 2 and Scheme 3. Polymerization of OEGA proceeded in 65% yield. The molecular characteristics of PnBA and PnBA-b-POEGA copolymers determined by SEC and ^1^H-NMR spectroscopy are provided in Table 1. The obtained M_w_ were in agreement with the calculated stoichiometric ones. The M_w_/M_n_ values, reported in Table 1, fall within the range usually reported for RAFT synthesized homopolymers and block copolymers. A representative SEC chromatogram of 4000 g/mol PnBA and PnBA_30_-b-POEGA_70_ diblock is depicted in Figure 1, highlighting the increase in M_w_ after the addition of POEGA. The M_w_/M_n_ are narrow, monomodal, and almost symmetrical, with a minimal number of tails appearing at high elution volumes (in the case of PnBA_30_-b-POEGA_70_ diblock), indicating an almost integrated reinitiation of RAFT polymerization and a nearly clean extension of the block sequence.

**Figure 2:**
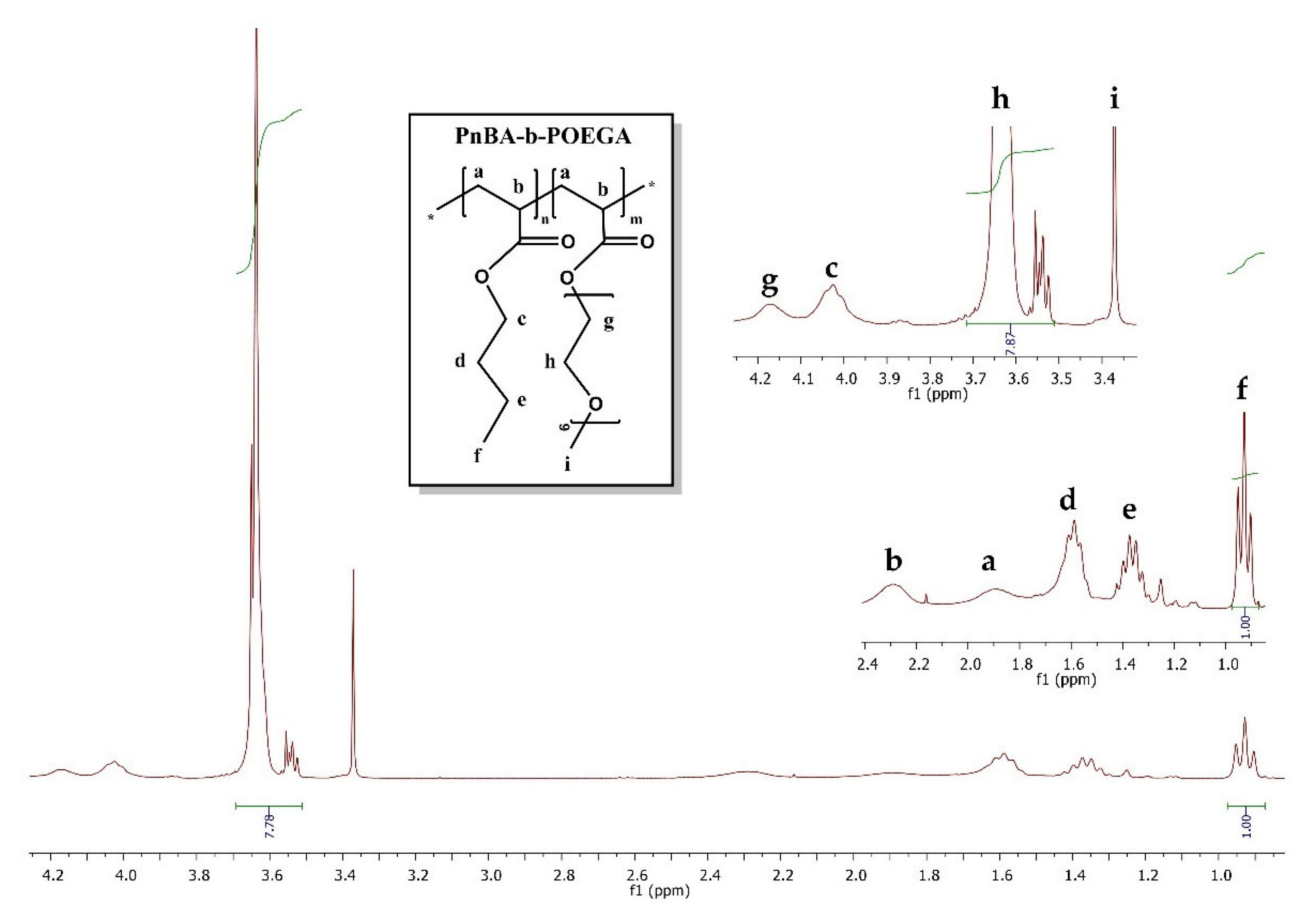
^1^H-NMR spectrum for the PnBA_30_-b-POEGA_70_ block copolymer in CDCl_3,_ where the black letters denote the protons of the copolymer structure (**a**–**i**). Insets are expansions of selected regions of the spectrum.

The compositions of PnBA-b-POEGA diblock copolymers were verified by ^1^H-NMR spectroscopy. A representative ^1^H-NMR spectrum of the PnBA_30_-b-POEGA_70_ diblock is presented in Figure 2. The calculated composition values are also summarized in Table 1. All data manifest a successful and controlled polymerization process.

### 3.2. Physicochemical Characterization of the PnBA-b-POEGA Micelles

Detailed physicochemical characterization is provided for the assessment of CMC, size, shape, surface charge, and chemical structure of PnBA-b-POEGA block copolymers by means of FS, DLS, SLS, ELS, and ATR-FTIR techniques. CMC was determined using the FS technique by entrapping Py (fluorescence probe) into the micellar core of PnBA-b-POEGA copolymers [see Section S1.1, Figure S1, Supplementary Materials]. Both CMC values are listed in Table 2.

**Figure 3:**
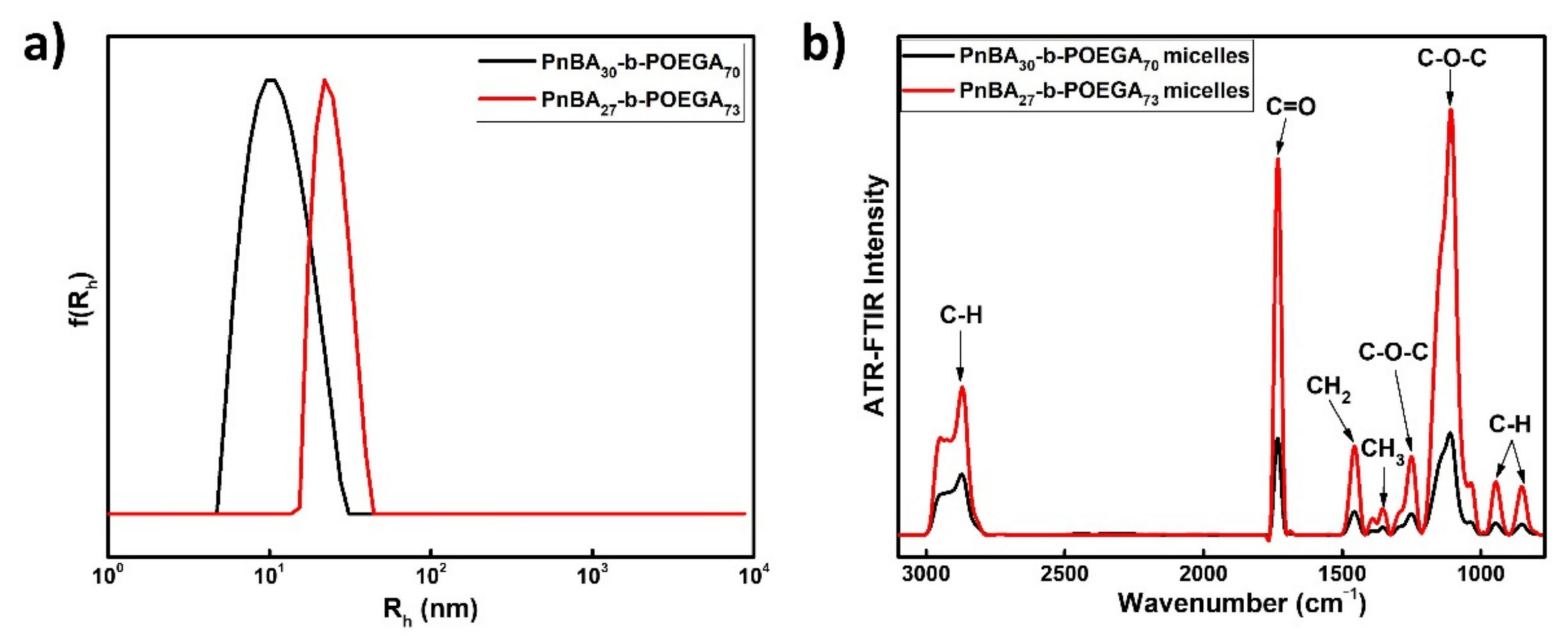
Comparative (**a**) size distributions from CONTIN of PnBA_30_-b-POEGA_70_ and PnBA_27_-b-POEGA_73_ copolymers at 90° in aqueous solutions using the TFHM, (**b**) attenuated total reflection Fourier transform (ATR-FTIR) spectra of PnBA-b-POEGA micelles in aqueous solutions using the TFHM.

Based on the chain architecture and DLS/SLS data, the novel diblocks self-assembled into nanosized core-shell micelles in aqueous media with PnBA hydrophobic cores and a hydrophilic POEGA corona. A comparison of intensity weighted and monomodal size distribution plots of PnBA_30_-b-POEGA_70_ and PnBA_27_-b-POEGA_73_ diblocks is presented in Figure 3a. The two symmetrical peaks indicate the participation of all chains in the formation of block copolymer micelles. Higher M_w_ of PnBA elicited an important growth in hydrodynamic radius (R_h_), an almost sixfold higher scattered light intensity value (mass) and a rather increased aggregation number (N_agg,_ obtained by SLS) of PnBA_27_-b-POEGA_73_ diblock (red line) compared to PnBA_30_-b-POEGA_70_ (black line), introducing an increase in the total M_w_ of the system. The values of R_h_, scattered light intensity, polydispersity index (PDI), and N_agg_ of the PnBA-b-POEGA diblock copolymers formed in aqueous media at C = 10^−3^ g/mL, pH = 7, and 25 °C are summarized in Table 2. ELS measurements of the PnBA-b-POEGA copolymers in aqueous media revealed slightly negative ζ_pot_, as seen in Table 2, maybe due to the presence of carboxylic acid chain end groups from the CTA.

Chain length (L) values of the PnBA-b-POEGA diblock copolymers are also displayed in Table 2, as a measure of particle dimensions. The low values of L are in agreement with the R_h_ values obtained from DLS measurements, underlying the spherical core-shell morphology of the PnBA_27_-b-POEGA_73_ micelles and verifying the increase in total M_w_.

ATR-FTIR measurements confirmed the chemical structure of the PnBA-b-POEGA micelles. Figure 3b exhibits the ATR-FTIR spectra of PnBA_30_-b-POEGA_70_ and PnBA_27_-b-POEGA_73_ micelles in aqueous media. Both spectra present the characteristic absorption bands due to the stretching vibrations of aliphatic C–H at the 3000–2770 cm^−1^ region; C=O stretching vibration at 1730 cm^−1^; −CH_2_ bending vibrations at 1455 cm^−1^, 945 cm^−1^, and 850 cm^−1^; −CH_3_ bending vibrations at 1355 cm^−1^; stretching vibrations of C–O–C at 1250 cm^−1^; and C–O stretching vibrations at 1107 cm^−1^.

### 3.3. FBS Interactions with PnBA-b-POEGA Block Copolymers

**Figure 4:**
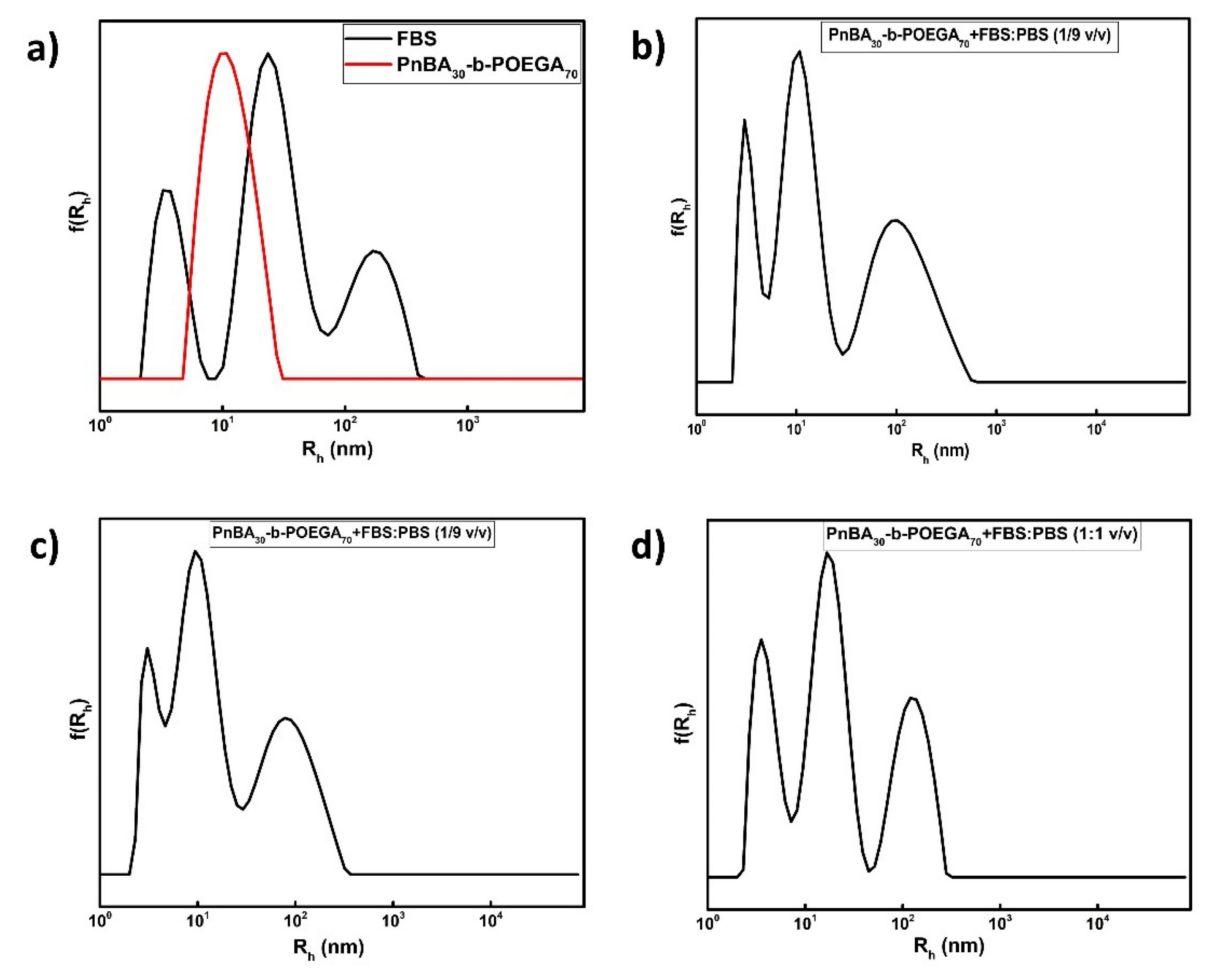
(**a**) Comparative size distributions of bare PnBA_30_-b-POEGA_70_ micelles and FBS (fetal bovine serum), (**b**) intensity size distributions of PnBA_30_-b-POEGA_70_ + FBS:PBS (1/9 *v*/*v*) using protocol 1, (**c**) size distributions of PnBA_30_-b-POEGA_70_ + FBS:PBS (1/9 *v*/*v*) using protocol 2, and (**d**) size distributions of PnBA_30_-b-POEGA_70_ + FBS:PBS (1/1 *v*/*v*) using protocol 2.

The lack of stability of polymeric biomaterials in a biological environment could induce dramatic changes in their synthetic identity as a result of recurring chemical/physical interactions between the polymer and the medium components. Thus, DLS stability studies of amphiphilic PnBA-b-POEGA micelles in FBS solutions were performed to detect possible alterations of their physicochemical characteristics in terms of size, PDI, and scattered light intensity. FBS simulates the physicochemical conditions of blood, and contains a variety of proteins, including a substantial amount (approximately 2.5 mg/mL) of bovine serum albumin (BSA). It is considered to differentiate/elucidate the properties of the micelles when injected into the human plasma [21]. A comparison of intensity size distributions of bare PnBA_30_-b-POEGA_70_ (red line) micelles and FBS (black line) is presented in Figure 4a. Protocol 1 [mixing of 50 μL sample with 3 mL FBS:PBS (1/9 *v*/*v*)] used for the preparation of protein–polymer mixtures is denoted in Figure 4b, whereas protocol 2 [mixing of 100 μL sample with 3 mL FBS:PBS (1/9 *v*/*v*) and 3 mL FBS:PBS (1/1 *v*/*v*)] is denoted in Figure 4c,d. The physicochemical characteristics (R_h_, PDI, Intensity) of FBS and protein–polymer mixtures with respect to both protocols are summarized in Table 3. The great heterogeneity of proteins in FBS elicited peculiar trimodal size distributions with peaks at about 4 nm, 23 nm, and 170 nm (Figure 4a, black line), underlying the existence of BSA at low values of R_h_ and larger size aggregates, respectively. The presence of characteristic FBS peaks in most DLS distributions is attributed to unbound proteins. After 1 h of PnBA_30_-b-POEGA_70_ incubation with FBS, DLS stability studies evidenced a coexistence of free proteins and PnBA_30_-b-POEGA_70_ micelles (Figure 4b–d). No major interactions between PnBA_30_-b-POEGA_70_ micelles and FBS (for both protocols) were reported, as the overall size (R_h_) of the micelles remained almost constant (10 nm) before and after mixing with serum proteins. The DLS stability studies of PnBA_27_-b-POEGA_73_ micelles in FBS solutions are reported in Section S1.2, Figure S2, Supplementary Materials, whereas their physicochemical characteristics are summarized in Table 3.

In terms of scattered light intensity (Table 3), a significant decrease in FBS is noticed compared to the protein–polymer mixtures using both protocols [except for protocol 2 at FBS:PBS (1/1 *v*/*v*) ratio], implying a mass shrinkage of FBS aggregates in most cases due to the presence of the polymer. Indeed, the R_h_ of the third population of FBS at 170 nm (Figure 4a) reduced by more than half after mixing with PnBA_30_-b-POEGA_70_ and PnBA_27_-b-POEGA_73_ micelles.

**Scheme 4:**
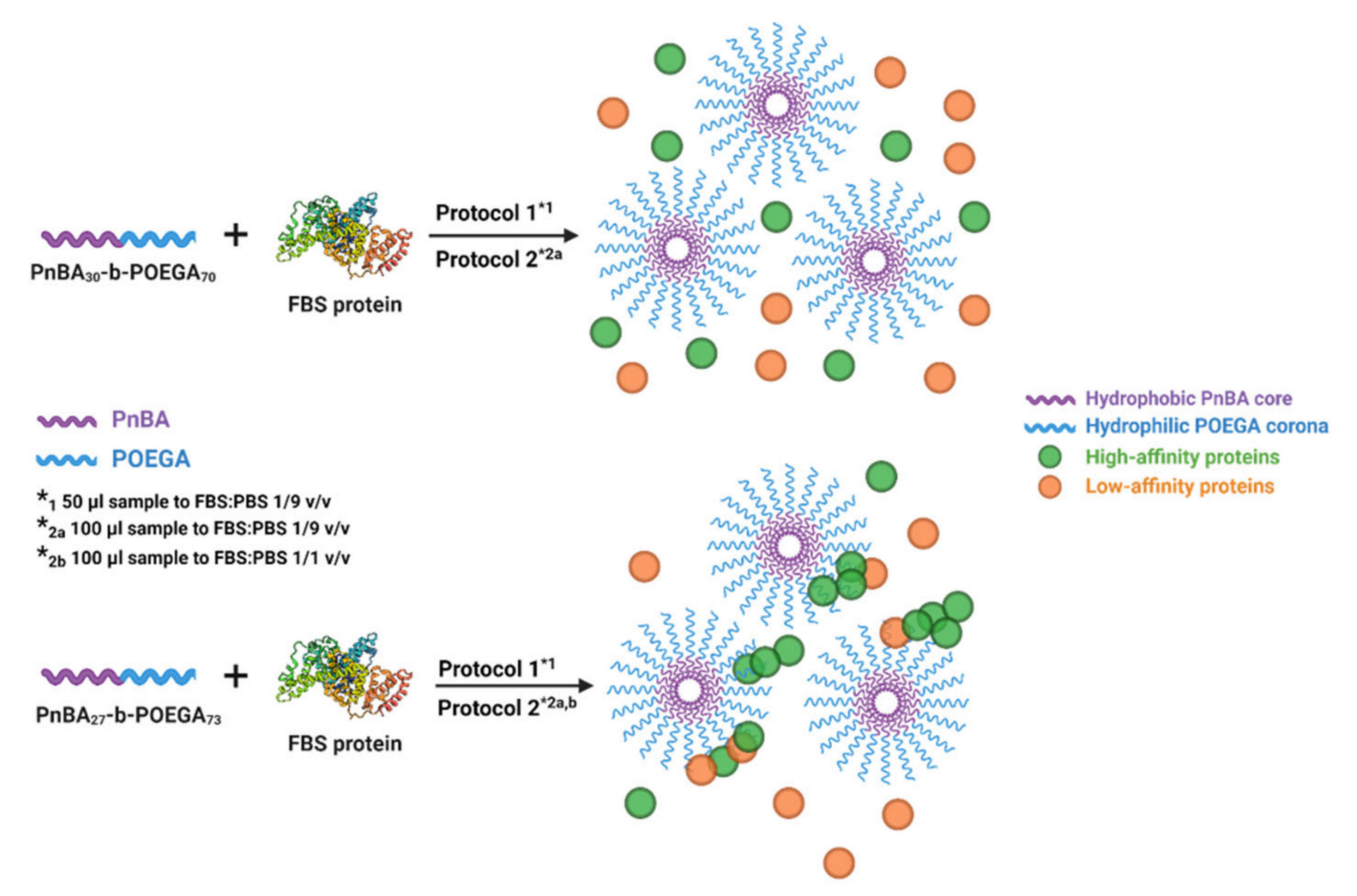
Schematic illustration of the interactions between PnBA_30_-b-POEGA_70_ and PnBA_27_-b-POEGA_73_ micelles and serum proteins using the two protocols (different FBS concentration in the mixed solutions).

All data manifested weak interactions between FBS and PnBA_30_-b-POEGA_70_ micelles. Collectively, DLS observations support the idea that POEGA hydrophilic chains shield PnBA_30_-b-POEGA_70_ and PnBA_27_-b-POEGA_73_ micelles (described in SI) against massive protein adsorption and particle agglomeration. A schematic illustration in Scheme 4 highlights the interactions between PnBA_30_-b-POEGA_70_ and PnBA_27_-b-POEGA_73_ micelles and serum proteins using the two protocols.

### 3.4. Physicochemical Characterization of the LSR-Loaded PnBA-b-POEGA Nanocarriers

**Figure 5:**
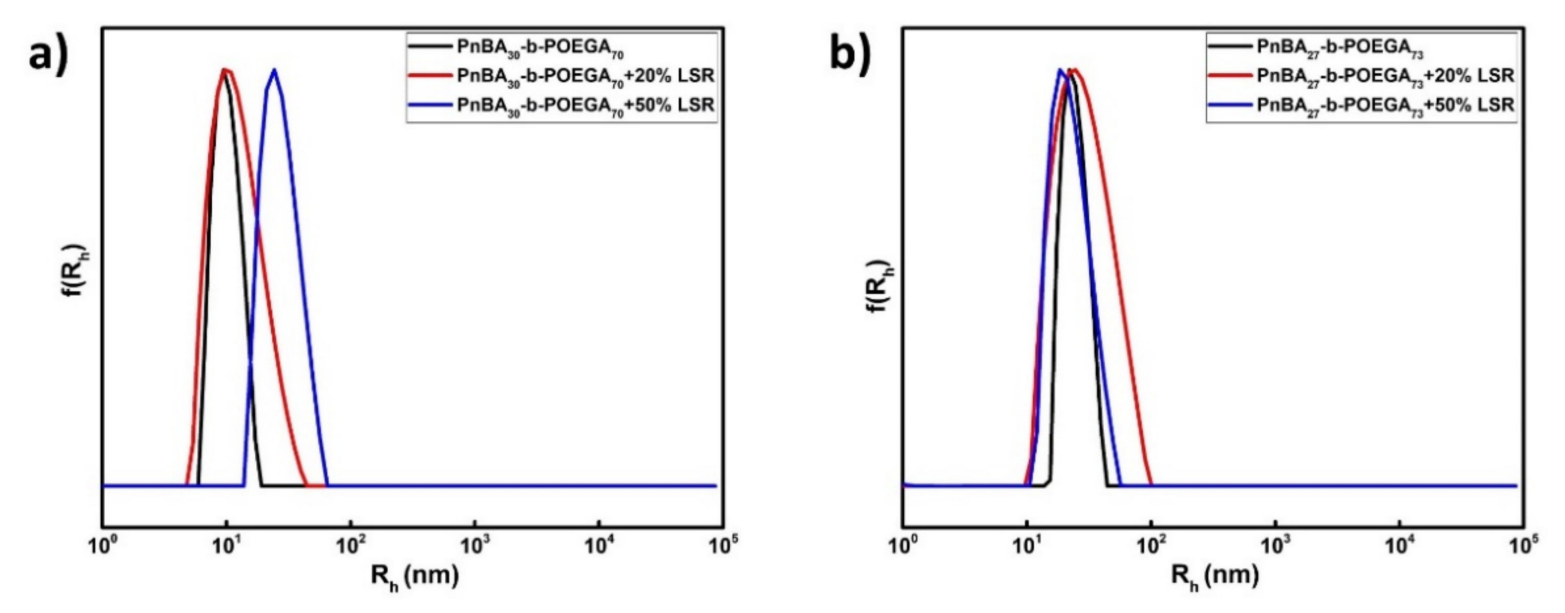
Comparative size distributions from CONTIN analysis at 90° for the (**a**) PnBA_30_-b-POEGA_70_ micelles, PnBA_30_-b-POEGA_70_ + 20% LSR and PnBA_30_-b-POEGA_70_ + 50% LSR nanocarriers, (**b**) PnBA_27_-b-POEGA_73_ micelles, PnBA_27_-b-POEGA_73_ + 20% LSR and PnBA_27_-b-POEGA_73_ + 50% LSR nanocarriers in aqueous solutions using the TFHM.

DLS, ELS, ATR-FTIR, and UV-Vis studies were carried out to determine the physicochemical properties of the LSR-loaded PnBA-b-POEGA nanocarriers, as well as to examine drug encapsulation into the polymeric PnBA micellar cores. The measurements were performed at 10^−3^ g/mL copolymer concentrations, pH = 7, and 25 °C. A comparison of size distributions of neat PnBA_30_-b-POEGA_70_/PnBA_27_-b-POEGA_73_ micelles and LSR-loaded PnBA-b-POEGA nanocarriers prepared by the TFHM is provided in Figure 5. All data revealed symmetrical and monomodal distributions for all solutions, indicating the participation of all chains in the formation of micellar nanostructures. Apparently, after LSR encapsulation into PnBA_30_-b-POEGA_70_ and PnBA_27_-b-POEGA_73_ micelles, slightly broader size distributions were found at 20 wt.% and 50 wt.% drug concentrations, probably due to the expected increase in the hydrophobic content of the mixed/drug-loaded nanocarriers. In addition, DLS data evidenced a significant increase in size (R_h_) and mass (based on determined scattered intensity from the solutions studied) of the LSR-loaded PnBA_30_-b-POEGA_70_ nanocarriers, indicating that the engulfing of LSR into the inner hydrophobic segments contributes to the formation of greater size drug–polymer nanostructures. DLS data highlight few detectable changes in the structural features of LSR-loaded PnBA-b-POEGA nanocarriers in comparison with the empty ones. Specifically, a gradual increase in the size and mass of PnBA_30_-b-POEGA_70_ + 20% LSR and PnBA_30_-b-POEGA_70_ + 50% LSR nanocarriers was observed (Table 4), implying the successful encapsulation of LSR into the PnBA_30_-b-POEGA_70_ polymeric micelles. The formation of lower mass and larger nanostructures is detected in the case of PnBA_27_-b-POEGA_73_ micelles loaded with 20 wt.% LSR. As LSR concentration increased to 50%, even lower size aggregates were observed compared to bare PnBA_27_-b-POEGA_73_ micelles. Yet, the existence of LSR increased the PDI value of the PnBA_27_-b-POEGA_73_ + 50% LSR nanocarriers.

ELS measurements displayed strongly negative ζ_pot_ values (Table 4) as LSR concentration increased from 20% to 50% for both PnBA_30_-b-POEGA_70_ and PnBA_27_-b-POEGA_73_ micelles due to the negative charge of the encapsulated LSR. The values of R_h_, scattered light intensity, PDI, and ζ_pot_ of the LSR-loaded PnBA-b-POEGA nanocarriers formed by TFHM in aqueous media are summarized in Table 4.

**Figure 6:**
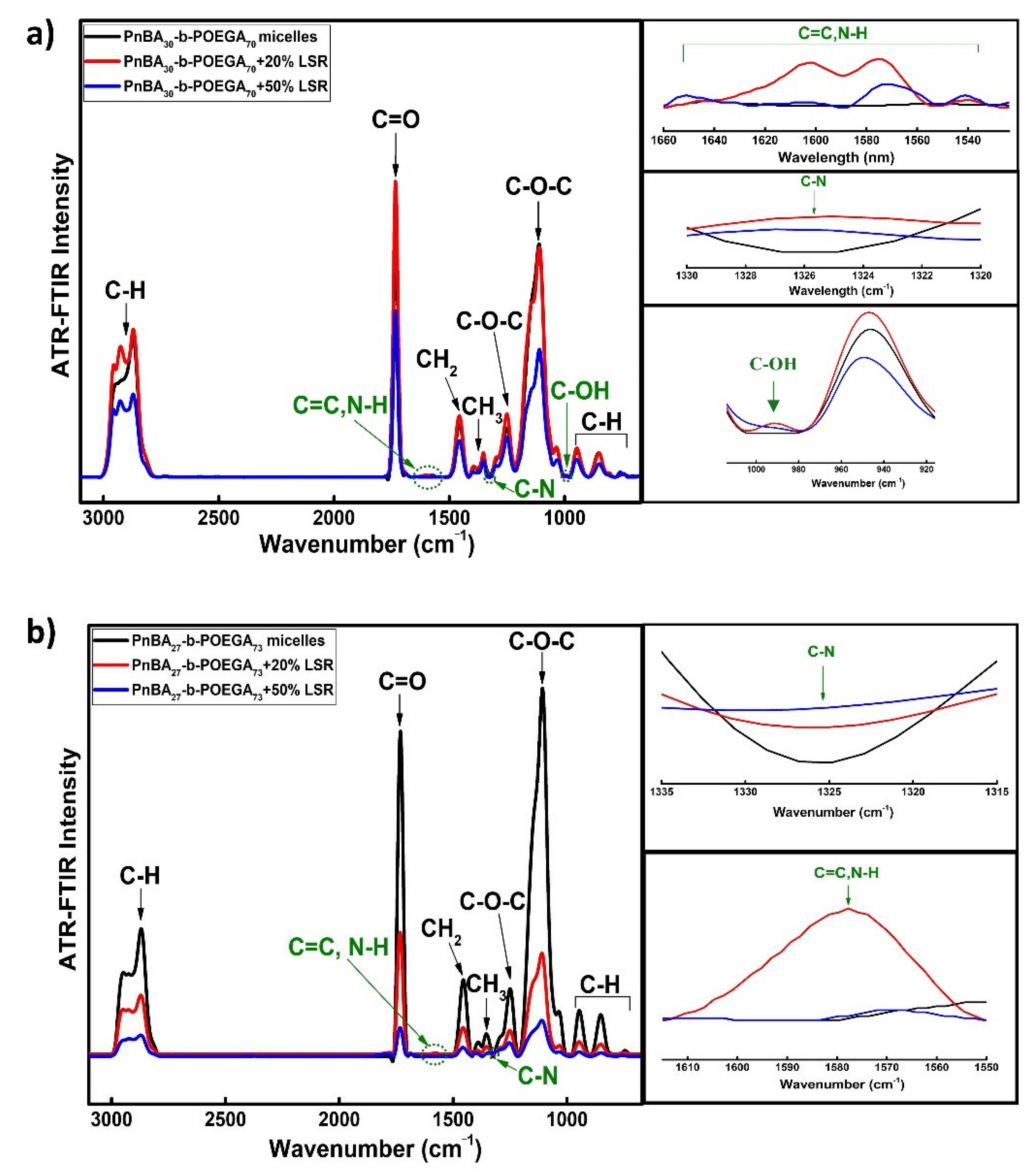
(**a**) Comparative ATR-FTIR spectra for the PnBA_30_-b-POEGA_70_ micelles, PnBA_30_-b-POEGA_70_ + 20% LSR and PnBA_30_-b-POEGA_70_ + 50% LSR nanocarriers, (**b**) comparative ATR-FITR spectra for the PnBA_27_-b-POEGA_73_ micelles, PnBA_27_-b-POEGA_73_ + 20% LSR, and PnBA_27_-b-POEGA_73_ + 50% LSR nanocarriers in aqueous solutions using the TFHM.

ATR-FTIR and UV-Vis measurements were pursued to examine LSR encapsulation into the polymeric core of the PnBA-b-POEGA micelles. A comparison of ATR-FTIR of neat PnBA_30_-b-POEGA_70_/PnBA_27_-b-POEGA_73_ micelles and LSR-loaded PnBA-b-POEGA nanocarriers prepared by TFHM is provided in Figure 6a,b. A critical decline in ATR-FTIR intensity peaks is noticed after 20% and 50% LSR encapsulation, denoting significant structural changes in both copolymer–drug systems. Likewise, new characteristic peaks of LSR (green annotations) are clearly observed in the ATR-FTIR spectra of the nanocarriers. In both Figure 6a,b, the appearance of new characteristic absorption peaks at 1500–1650 cm^−1^ are correlated to C=C stretching modes, which are attributed to the phenyl rings and secondary amine N–H bending vibrations of LSR. Moreover, the absorption peaks at 1319–1330 cm^−1^ are probably related to C–N stretching vibrations of the LSR aromatic ring. The PnBA_30_-b-POEGA_70_ + 20%LSR and PnBA_30_-b-POEGA_70_ + 50%LSR nanocarriers of Figure 6a introduce an extra absorption peak at 1000–1075 cm^−1^ compared to PnBA_27_-b-POEGA_73_ + 20%LSR and PnBA_27_-b-POEGA_73_ + 50%LSR of Figure 6b, which is attributed to the primary OH stretching vibrations of LSR [44,45]. All data confirm the existence of LSR in the mixed copolymer–drug aqueous solutions.

**Figure 7:**
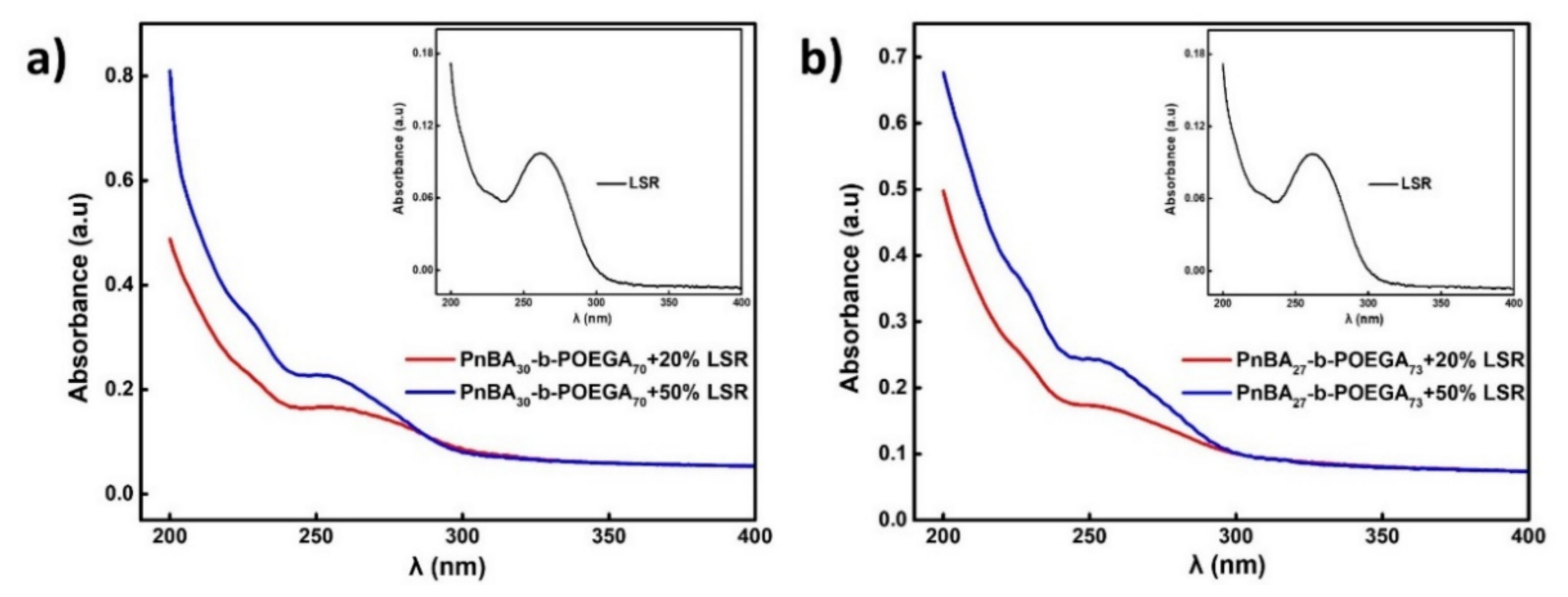
Comparative UV-Vis spectra for the (**a**) PnBA_30_-b-POEGA_70_ micelles, PnBA_30_-b-POEGA_70_ + 20% LSR, and PnBA_30_-b-POEGA_70_ + 50% LSR nanocarriers; and (**b**) PnBA_27_-b-POEGA_73_ micelles, PnBA_27_-b-POEGA_73_ + 20% LSR, and PnBA_27_-b-POEGA_73_ + 50% LSR nanocarriers in aqueous solutions prepared by the TFHM.

UV-Vis measurements further verified the successful encapsulation of LSR into the PnBA-b-POEGA polymeric micelles. Figure 7 presents the UV-Vis spectra of neat PnBA_30_-b-POEGA_70_/PnBA_27_-b-POEGA_73_ micelles and LSR-loaded PnBA-b-POEGA nanocarriers prepared by TFHM in aqueous solutions along with the corresponding spectra of LSR in ACTN. In line with the literature data, the UV spectrum of LSR exhibits maximum absorbance at 205 nm, 225 nm, and 254 nm wavelengths [44,46,47,48,49]. However, the UV-Vis spectra of LSR, depicted in Figure 7a,b (black line), exhibit two characteristic absorption peaks at 260 nm and 228 nm. Given that PnBA-b-POEGA diblock copolymers do not absorb at the respective wavelength range in the UV spectrum, the presence of the two UV absorption peaks (254 nm and 228 nm) of PnBA_30_-b-POEGA_70_ + 20% LSR/PnBA_30_-b-POEGA_70_ + 50% LSR (Figure 7a) and PnBA_27_-b-POEGA_73_ + 20% LSR/PnBA_27_-b-POEGA_73_ + 50% (Figure 7b) nanocarriers is attributed to the LSR UV absorption peaks. As LSR concentration increases to 50% (blue lines), an increase in the UV absorption of LSR at both wavelengths is clearly evident, highlighting a greater/stronger LSR encapsulation into the micelles. Notably, the detected shifting in the peak maxima may be related to hydrophobic and hydrogen bonding copolymers/drug interactions.

### 3.5. Stability Studies of LSR-Loaded PnBA-b-POEGA Nanocarriers

**Figure 8:**
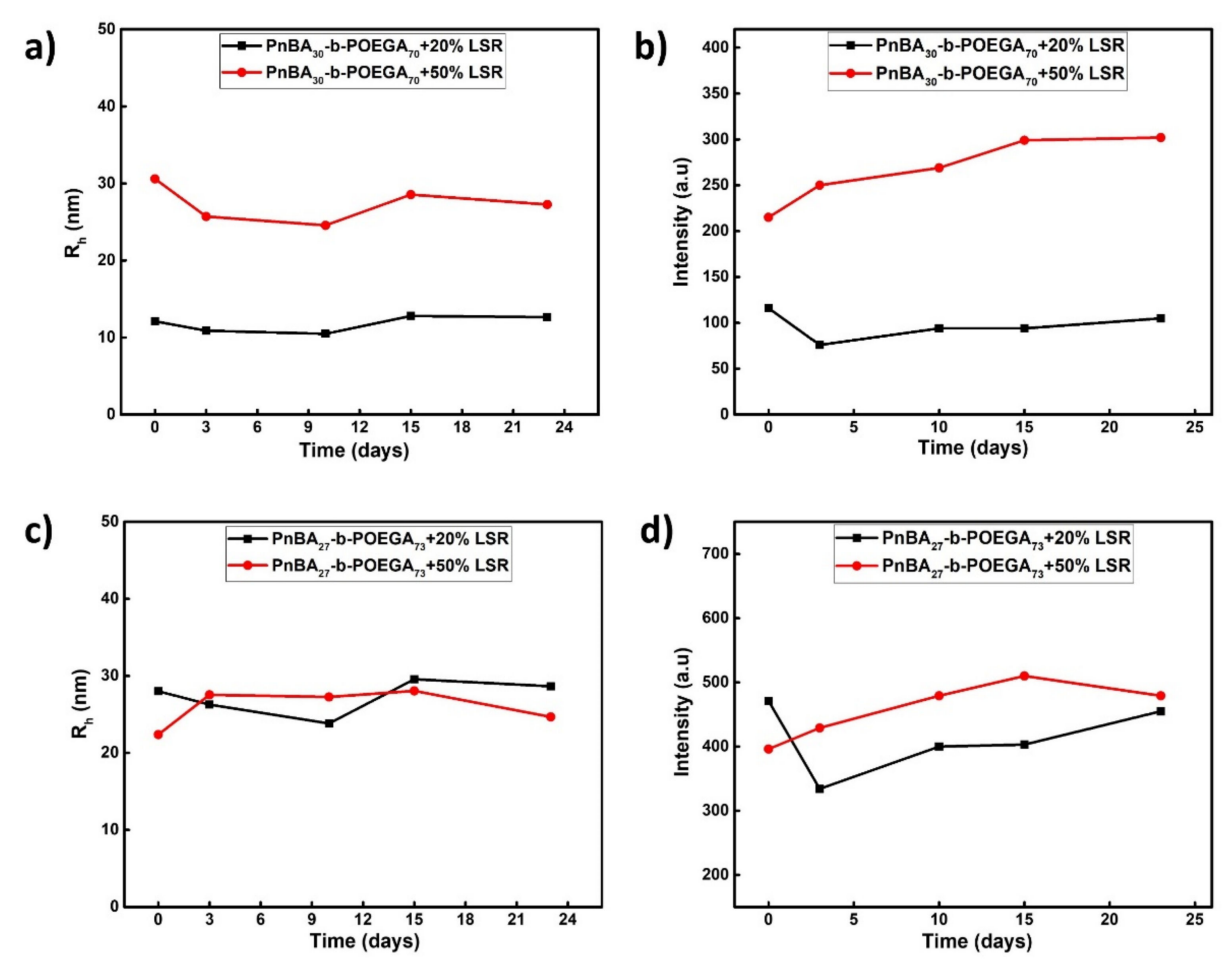
R_h_ and scattered intensity measurements versus time obtained from DLS of (**a**,**b**) PnBA_30_-b-POEGA_70_ + 20% LSR and PnBA_30_-b-POEGA_70_ + 50% LSR nanocarriers, and (**c**,**d**) PnBA_27_-b-POEGA_73_ + 20% LSR and PnBA_27_-b-POEGA_73_ + 50% LSR nanocarriers in aqueous solutions using the TFHM.

Stability studies were performed using DLS to estimate the temporal stability of the resulting LSR-loaded PnBA-b-POEGA solutions over a period of 23 days. The R_h_ and scattered intensity measurements versus time (average of three measurements) of LSR-loaded PnBA-b-POEGA nanocarriers are depicted in Figure 8. DLS studies on the mixed-drug solutions verified their stability in aqueous solutions in terms of size and mass. Specifically, the PnBA_30_-b-POEGA_70_ + 20% LSR/PnBA_27_-b-POEGA_73_ + 50% LSR nanocarriers (Figure 8a,b), as well as the PnBA_27_-b-POEGA_73_ + 20% LSR/PnBA_27_-b-POEGA_73_ + 50% LSR (Figure 8c,d), do not exhibit significant R_h_ and intensity fluctuations, indicating stable nanostructures. Slight variations in scattered light intensity are present in the case of PnBA_30_-b-POEGA_70_ + 50% LSR nanocarriers (Figure 8b), suggesting an appreciably stable drug–polymer system within 23 days. Remarkably, the mixed copolymer–drug solutions exhibited great long-term stability to the naked eye (approximately 2 years), as no visually detectable drug precipitation was observed.

### 3.6. ^1^H-NMR Studies on PnBA-b-POEGA Micelles and LSR-Loaded PnBA-b-POEGA Nanocarriers

**Figure 9:**
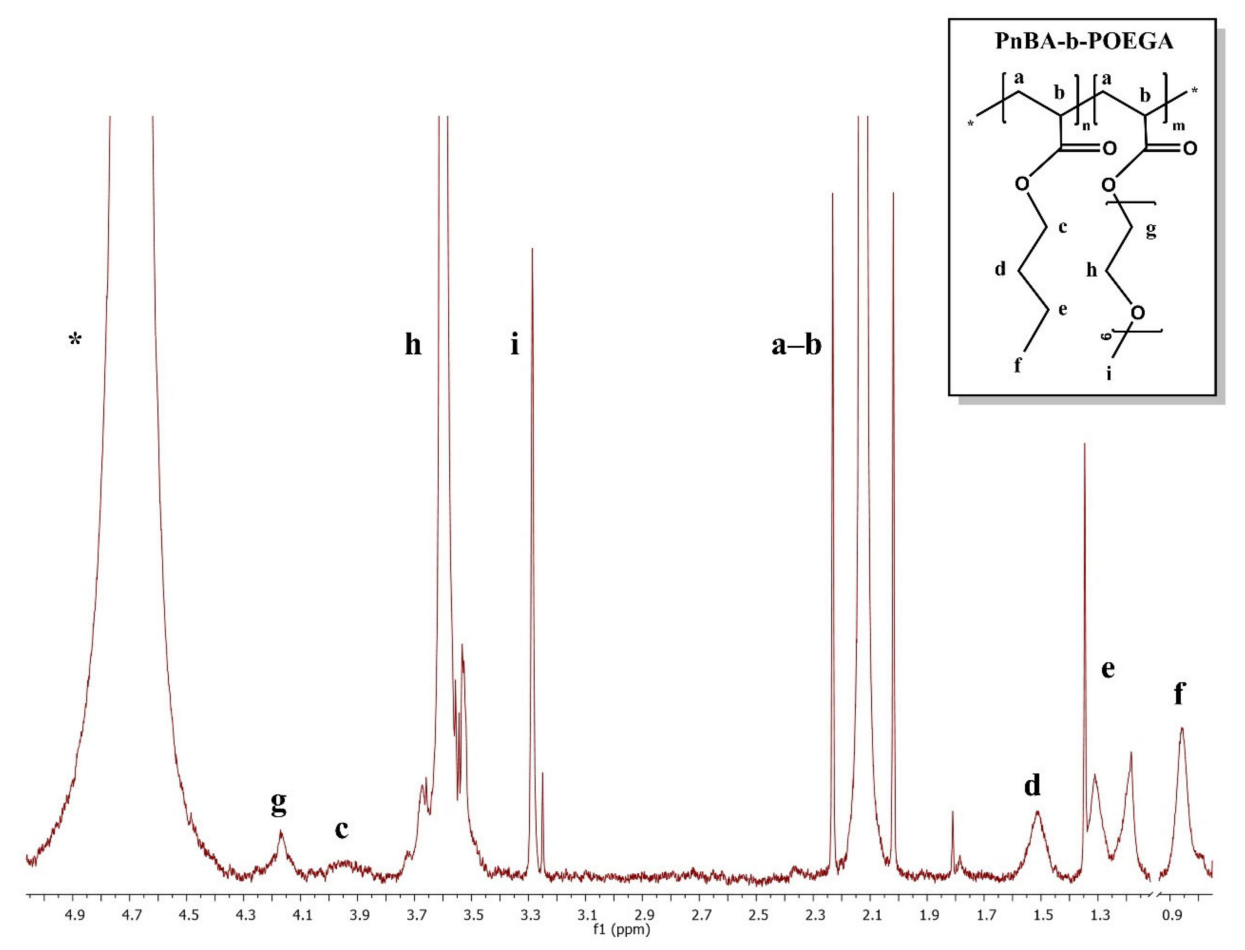
^1^H-NMR spectrum for the PnBA_30_-b-POEGA_70_ micelles in D_2_O solutions, where the black letters denote the protons of the copolymer structure (**a**–**i**) and the asterisk refers to the solvent D_2_O.

**Figure 10:**
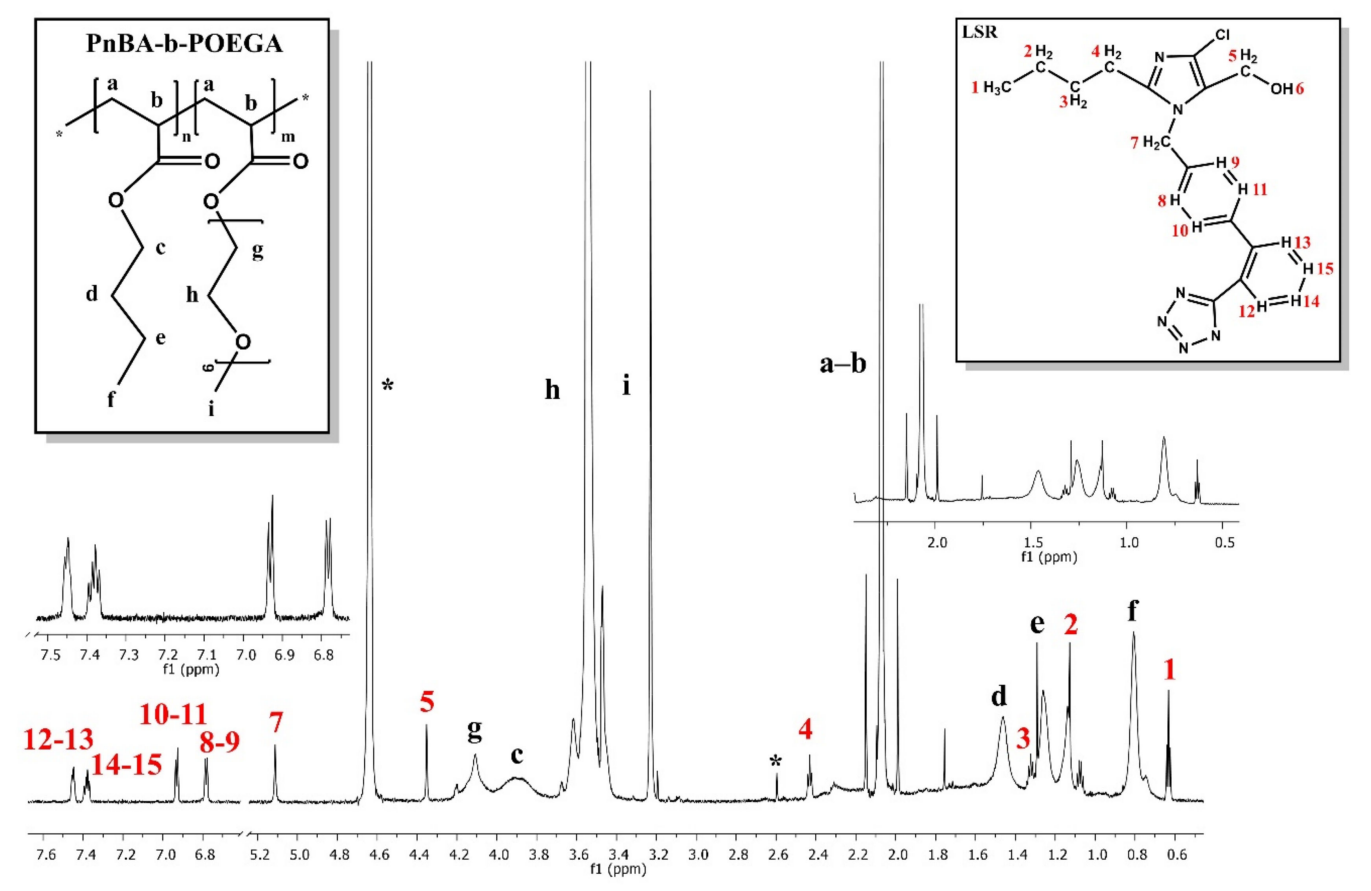
^1^H-NMR spectrum for the PnBA_30_-b-POEGA_70_ + 50% LSR nanocarriers in D_2_O solutions, where the black letters denote the protons of the copolymer structure (**a**–**i**), the red numbers point to the LSR peaks and the asterisk refers to the solvent D_2_O.

Additional ^1^H-NMR studies on PnBA_30_-b-POEGA_70_ micelles and PnBA_30_-b-POEGA_70_ + 50% LSR nanocarriers were carried out in D_2_O solutions to investigate the aggregation behavior of the micelles and confirm the presence of LSR in the drug–copolymer solutions. Structural identification of LSR has been recited earlier in the literature [44]. The ^1^H-NMR spectra for the PnBA_30_-b-POEGA_70_ micelles and PnBA_30_-b-POEGA_70_ + 50% LSR nanocarriers in D_2_O solutions are exhibited in Figure 9 and Figure 10. The black letters in both ^1^H-NMR spectra display the protons of the copolymer structure, whereas the red letters depicted in the ^1^H-NMR spectrum of PnBA_30_-b-POEGA_70_ + 50% LSR nanocarriers show the LSR peaks. The ^1^H-NMR spectrum of PnBA_30_-b-POEGA_70_ + 50% LSR nanocarriers displayed in Figure 10 shows the expected proton signals of LSR, verifying the successful loading of LSR into the polymeric nanocarriers. The chemical shifts of the PnBA_30_-b-POEGA_70_ proton signals in the absence (Figure 9) and in the presence of LSR (Figure 10) are summarized in Table 5. Additionally, the chemical shifts of LSR proton signals in the presence of PnBA_30_-b-POEGA_70_ copolymer and in sodium dodecyl sulfate (SDS) micelles are exhibited in Table 6, reflecting its great flexibility [32].

A shielding of the copolymer resonances is shown in the presence of LSR, evidencing the successful loading of LSR in the micellar environment. LSR chemical shifts are also driven to upfield values, reaching Δδ differences of up to 0.19 ppm, indicating its strong interactions with the copolymer structure.

### 3.7. 2D-COSY Studies on LSR-Loaded PnBA-b-POEGA Nanocarriers

**Figure 11:**
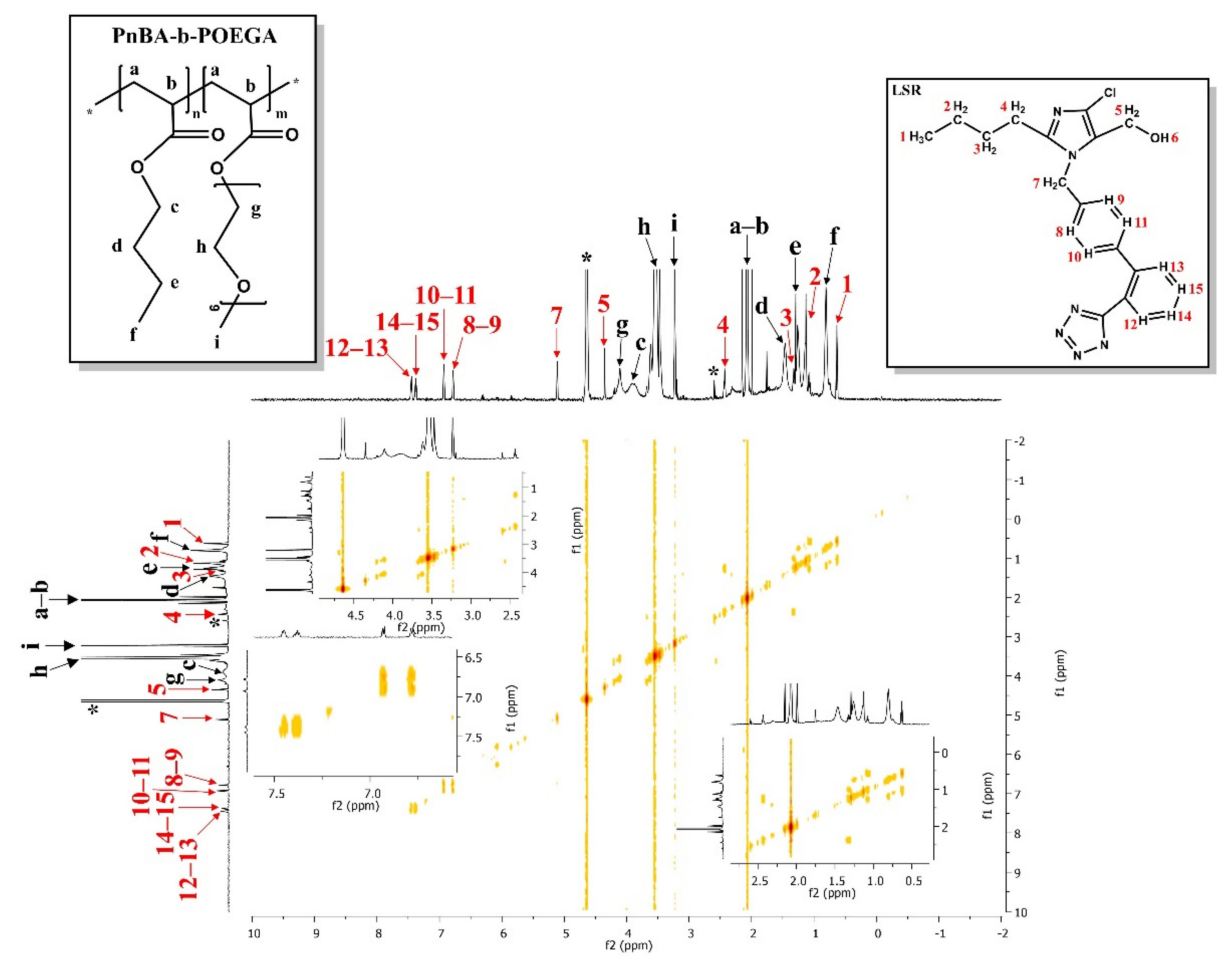
2D-COSY spectrum of PnBA_30_-b-POEGA_70_ + 50% LSR nanocarriers in D_2_O solutions, where the black letters (**a**–**i**) denote the protons of the copolymer structure, and the red numbers point to the LSR peaks. The asterisk refers to the solvent D_2_O.

The intramolecular interactions between the PnBA_30_-b-POEGA_70_ copolymer and LSR moieties and their internal structure were evaluated by 2D-COSY measurements. The 2D-COSY spectrum of the PnBA_30_-b-POEGA_70_ + 50% LSR nanocarriers is presented in Figure 11, where the black arrows denote the protons of the copolymer structure while the red ones point to the LSR peaks. The cross peaks, noticed between the protons of the copolymer and the protons of LSR in the 2D-COSY spectrum in Figure 11, signify the intramolecular interactions and the structural elucidation of both copolymer and LSR.

### 3.8. 2D-NOESY Studies on LSR-Loaded PnBA-b-POEGA Nanocarriers

**Figure 12:**
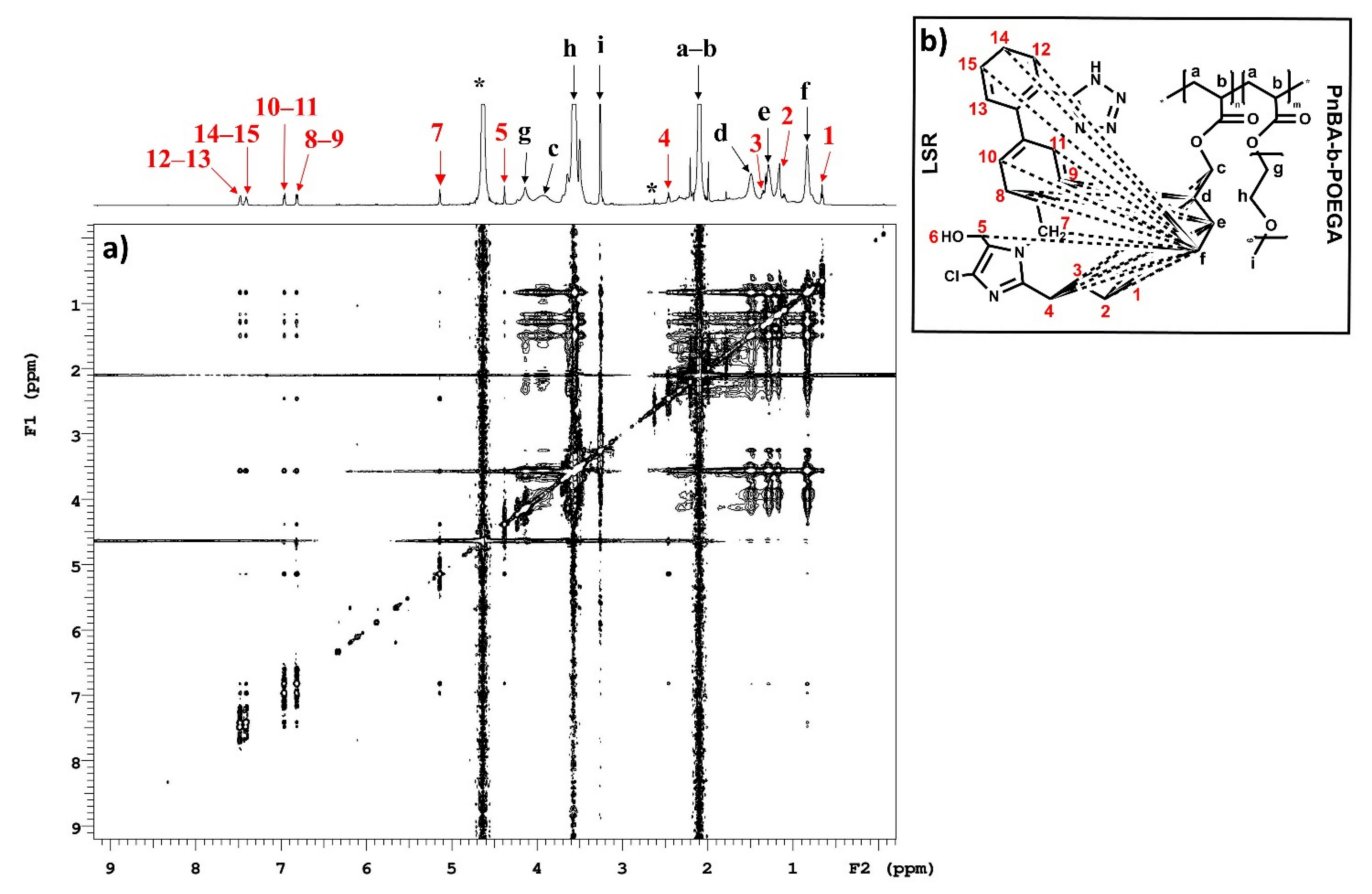
(**a**) 2D-NOESY spectrum of PnBA_30_-b-POEGA_70_ +50% LSR nanocarriers and (**b**) the schematic approach of LSR in PnBA_30_-b-POEGA_70_ micelles. The black letters (**a**–**i**) denote the protons of the copolymer structure, the red numberspoint to the LSR peaks, and the asterisk refers to the solvent D_2_O.

Τhe intramolecular/intermolecular interactions between the copolymer and LSR were further investigated via 2D-NOESY experiments for the verification of the successful loading of LSR into the polymeric PnBA_30_-b-POEGA_70_ micelles, and to study the spatial vicinities between the copolymer and LSR. The eminent correlations between PnBA_30_-b-POEGA_70_ and LSR moieties in D_2_O are juxtaposed in Figure 12. The cross peaks between the phenyl rings (8–9, 10–11, 12-13, 14–15) and butyl chain of LSR (1, 2, 3, 4) with the methylene signals of PnBA (c, d, e, f) are clearly evident (Figure 12), highlighting the strong binding of LSR with the PnBA hydrophobic core. Figure 12b illustrates the approach of LSR in PnBA_30_-b-POEGA_70_ micelles. Particularly, the correlations between the butyl chain (1, 2, 3, 4) of LSR and the butyl chain (c, d, e, f) of PnBA seem to be more eminent than those between the biphenyl ring (8–9, 10–11, 14–15, 12-13) of LSR and the butyl chain (c, d, e, f) of PnBA, evidencing that the hydrophobic interactions are mainly exerted by the butyl chain of LSR and the butyl chain of PnBA.

### 3.9. 2D-DOSY Studies on LSR-Loaded PnBA-b-POEGA Nanocarriers

**Figure 13:**
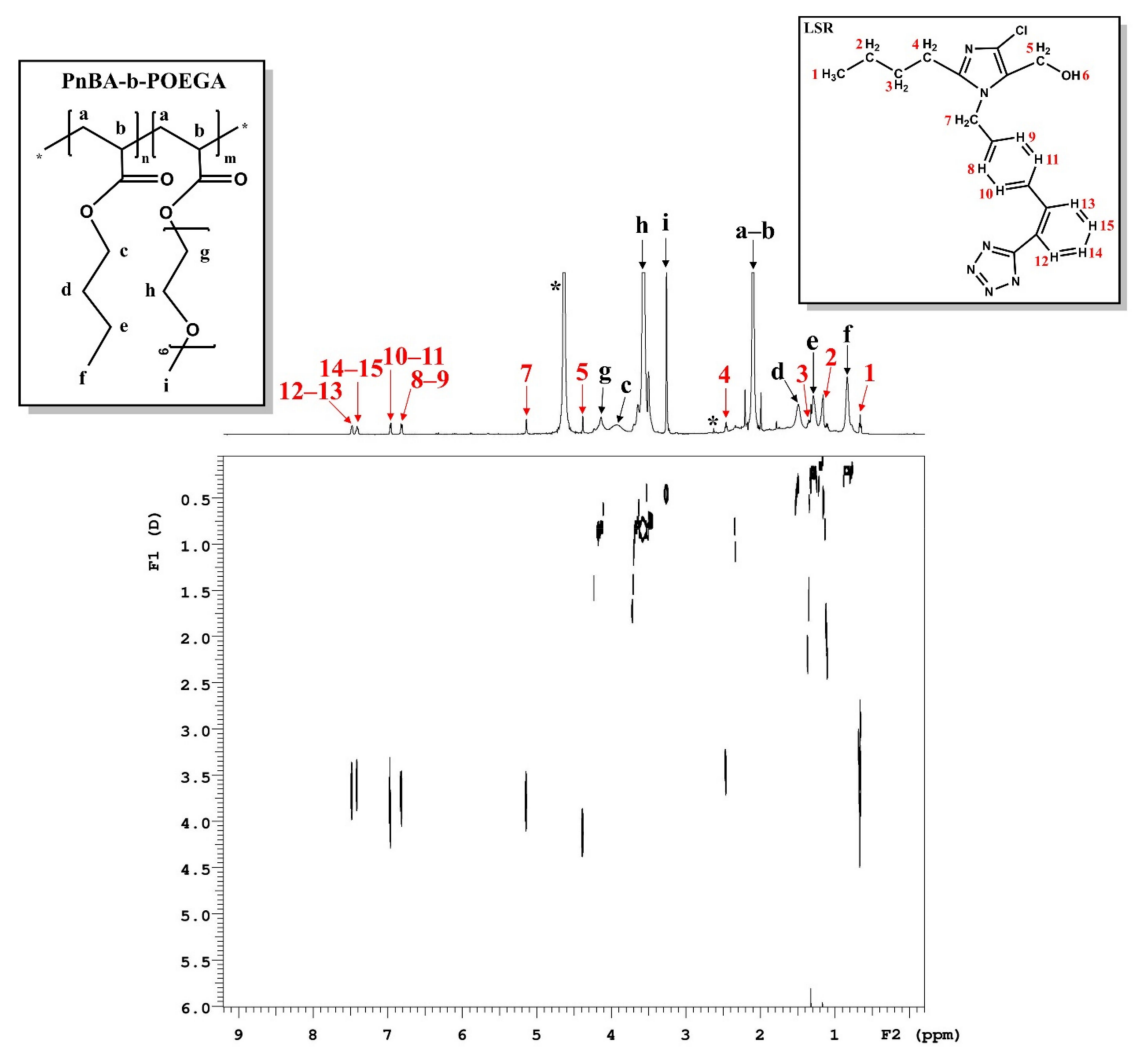
2D-DOSY spectrum of PnBA_30_-b-POEGA_70_ +50% LSR nanocarriers. The black letters (**a**–**i**) denote the protons of the copolymer structure, the red numbers point to the LSR peaks, and the asterisk refers to the solvent D_2_O.

2D-DOSY experiments were also performed for the determination of the self-diffusion coefficients D of the PnBA_30_-b-POEGA_70_ + 50% LSR nanocarriers and the identification of drug association with micelles and the presence of assemblies. Figure 13 indicates the diffusion experiments for the PnBA_30_-b-POEGA_70_ + 50% LSR nanocarriers to certify the association of LSR with PnBA_30_-b-POEGA_70_ micelles. Particularly, two series of traces with low (D = 0.27 × 10^−10^ m^2^ s^−1^) and medium constants (D = 0.83 × 10^−10^ m^2^ s^−1^) are shown in Figure 13, and may be related to the diffusions of block copolymer micelles and unimolecular block copolymers, respectively. The trace with the highest constant (D = 4.24 × 10^−10^ m^2^ s^−1^) depicted in Figure 13, may refer to the diffusion of LSR, suggesting a rather high exchange between the free and the micelle-bound state of LSR.

### 3.10. ^1^H-NMR Temperature Dependence Studies on POEGA Homopolymer and PnBA-b-POEGA Copolymer

**Figure 14:**
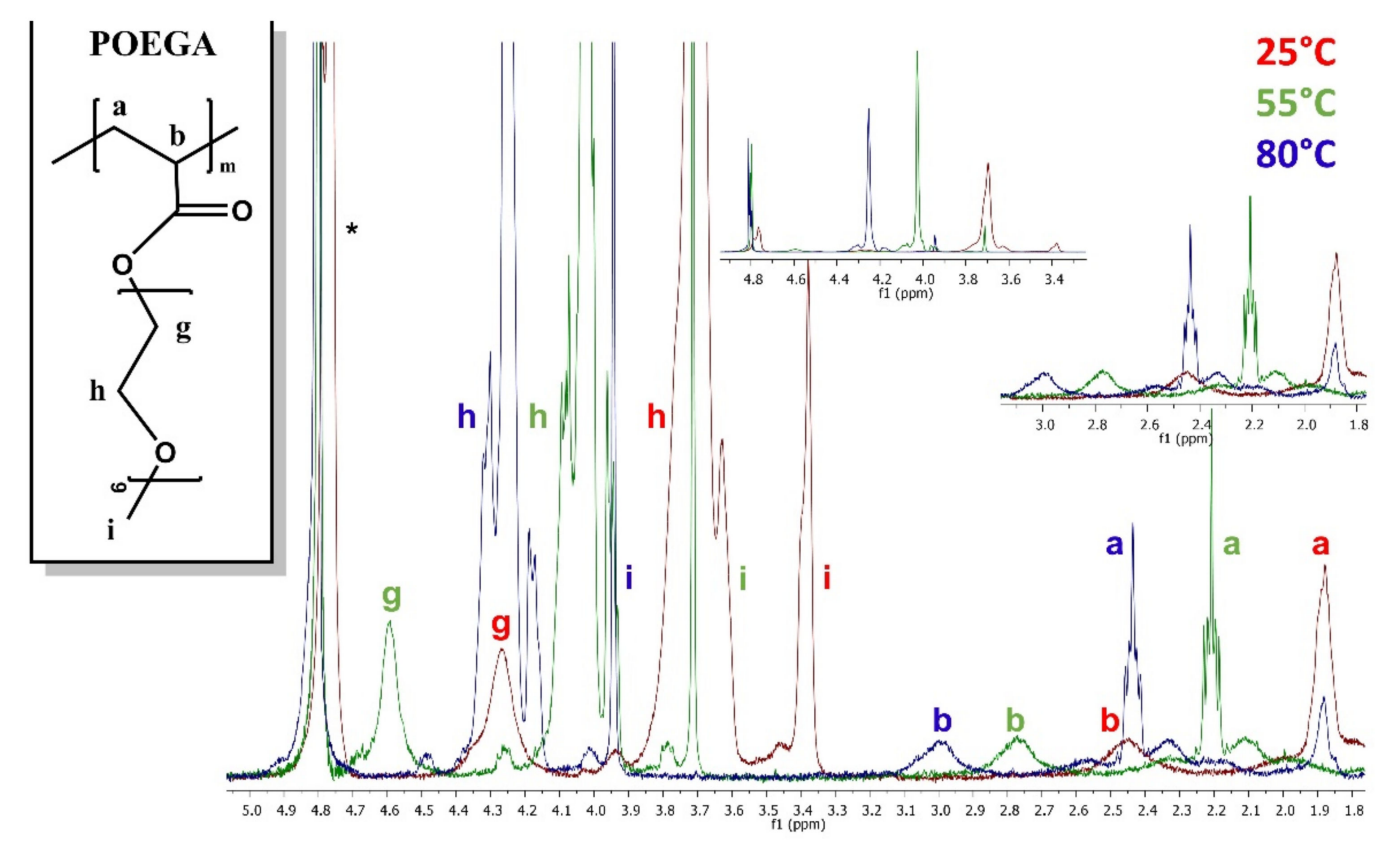
Comparison of ^1^H-NMR spectra of POEGA homopolymer at various temperatures, where the red, green, and blue letters refers to the protons of POEGA homopolymer at 25 °C, 55 °C, and 80 °C, respectively and the asterisk refers to the solvent D_2_O.

**Figure 15:**
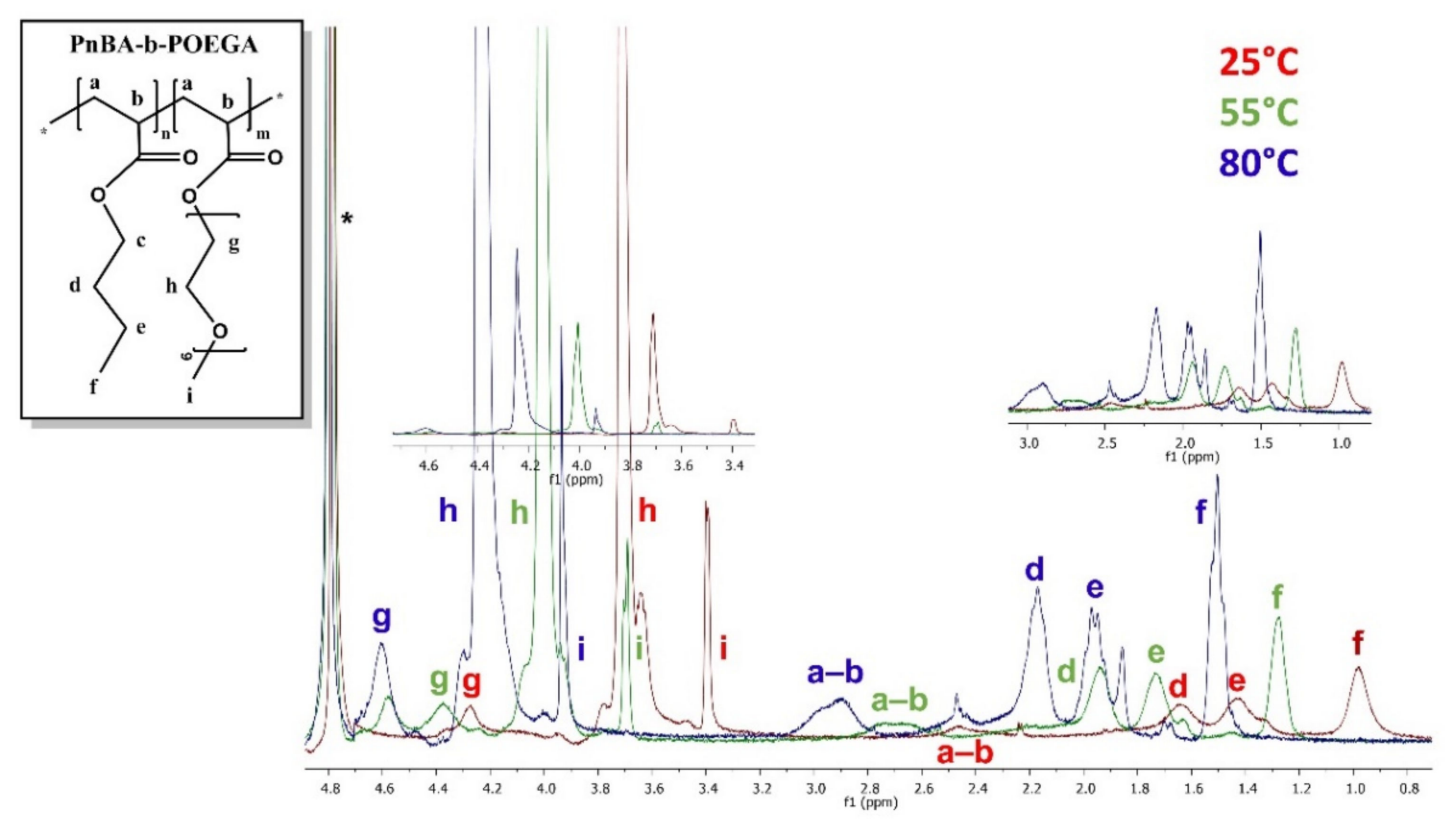
Comparison of ^1^H-NMR spectra of PnBA_30_-b-POEGA_70_ diblock at various temperatures. The red, green, and blue letters refer to the protons of PnBA_30_-b-POEGA_70_ copolymer at 25 °C, 55 °C, and 80 °C, respectively and the asterisk refers to the solvent D_2_O.

**Figure 16:**
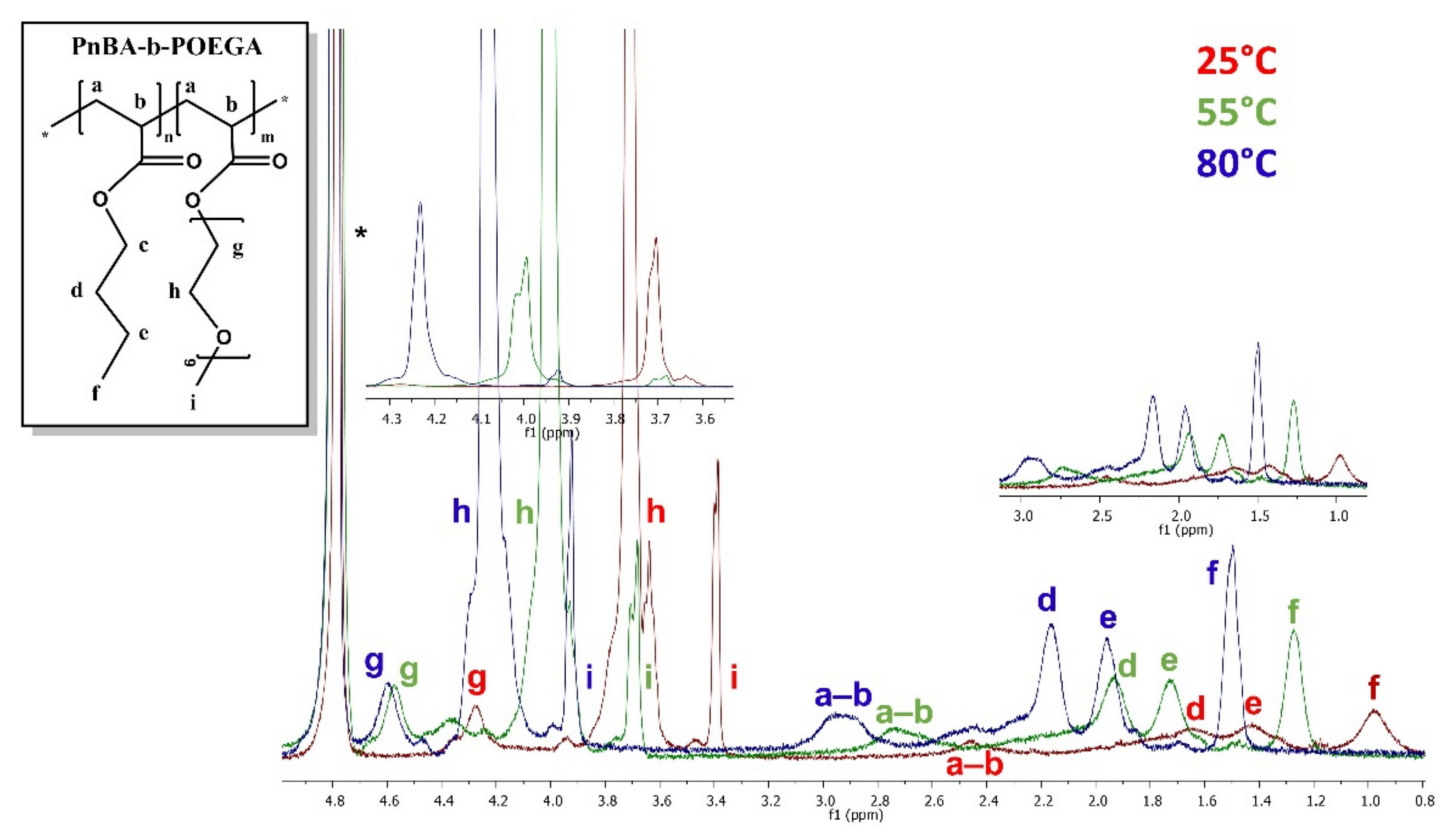
Comparison of ^1^H-NMR spectra of PnBA_27_-b-POEGA_73_ diblock at various temperatures. The red, green, and blue letters refer to the protons of PnBA_27_-b-POEGA_73_ copolymer at 25 °C, 55 °C, and 80 °C, respectively and the asterisk refers to the solvent D_2_O.

A systematic ^1^H-NMR study over a wide range of temperatures can reveal the mobility of protons during micellization, but, still, such studies are scarce in the literature. The ^1^H-NMR spectra of POEGA homopolymer, PnBA_30_-b-POEGA_70_, and PnBA_27_-b-POEGA_73_ diblock copolymers at three different temperatures are displayed in Figure 14, Figure 15 and Figure 16. Detectable fluctuations in the integrated intensity of the proton signals of POEGA are observed in the ^1^H-NMR spectrum (Figure 14) with increasing temperature, which is an indication of enhanced proton mobility. The half-width of POEGA is decreased, while the integrated intensity of the proton signals fluctuates from 25 °C to 80 °C. Specifically, the integrated intensity of the proton signals steeply raises as temperature increases from 25 °C to 55 °C. The subsequent decrease in the integrated intensity of the proton signals for temperatures from 55 °C to 80 °C signifies a small decrease in its solubility, which may be exerted by the rupture of hydrogen bonds upon heating. The ^1^H-NMR peaks of PnBA_30_-b-POEGA_70_ and PnBA_27_-b-POEGA_73_ diblock copolymers presented in Figure 15 and Figure 16 shifted with increasing temperature. At high temperatures (80 °C), the proton signals appear narrower and more intense, implying the enhanced mobility of the protons. The chemical shifting as a function of temperature of PnBA_30_-b-POEGA_70_ block copolymers is presented in Figure S4 in the Supplementary Materials to provide a clearer view of the shifting of the ^1^H-NMR spectra (Figure 15) to the lower field regions.

Apparently, the novel PnBA-b-POEGA copolymers are considered temperature-independent AmBCs, containing a biocompatible and rather hydrophobic easily deformable PnBA segment [50]. Based on the low T_g_ value (−54 °C) of PnBA [46,51], heating may trigger partial disintegration or increased fluidity of the polymer core matrix by inducing water penetration in the PnBA core, resulting in increased proton mobility. The effect of temperature on PnBA_30_-b-POEGA_70_ block copolymers was also investigated by DLS measurements to provide useful information of the overall hydrodynamic behavior of the globular structures. DLS data analysis details are presented in Section S1.3, Figure S3, Supplementary Materials.

### 3.11. Encapsulation and Ultrasound Release Studies

DL% and %EE were calculated by means of UV-Vis spectroscopy. The ultrasound-triggered release behavior of LSR from PnBA_30_-b-POEGA_70_ and PnBA_27_-b-POEGA_73_ micelles was also considered. The highly stable PnBA_30_-b-POEGA_70_ + 50% LSR and PnBA_27_-b-POEGA_73_ + 50% LSR nanocarriers were hand-picked for the investigation of release kinetics, mainly due to the increased concentration of hydrophobic LSR (50%). The %DL and %EE values were calculated using Equations (2) and (3), and are summarized in Table 7. The highest hydrophobicity of the PnBA_27_-b-POEGA_73_ micelles, emanating from the increased M_w_ of the PnBA block (7800), seems to favor the encapsulation of hydrophobic LSR into the micelles, resulting to a higher value of %EE compared to PnBA_30_-b-POEGA_70_ + 50% LSR nanocarriers (Table 7). Given that PnBA is not considered a highly hydrophobic polymer (taking also into account the low T_g_ value) [46] in combination with the low M_w_ of the PnBA block (4000), the PnBA_30_-b-POEGA_70_ + 50% LSR nanocarriers exhibited low %EE, implying an inefficient loading of LSR into PnBA_30_-b-POEGA_70_ micelles.

**Figure 17:**
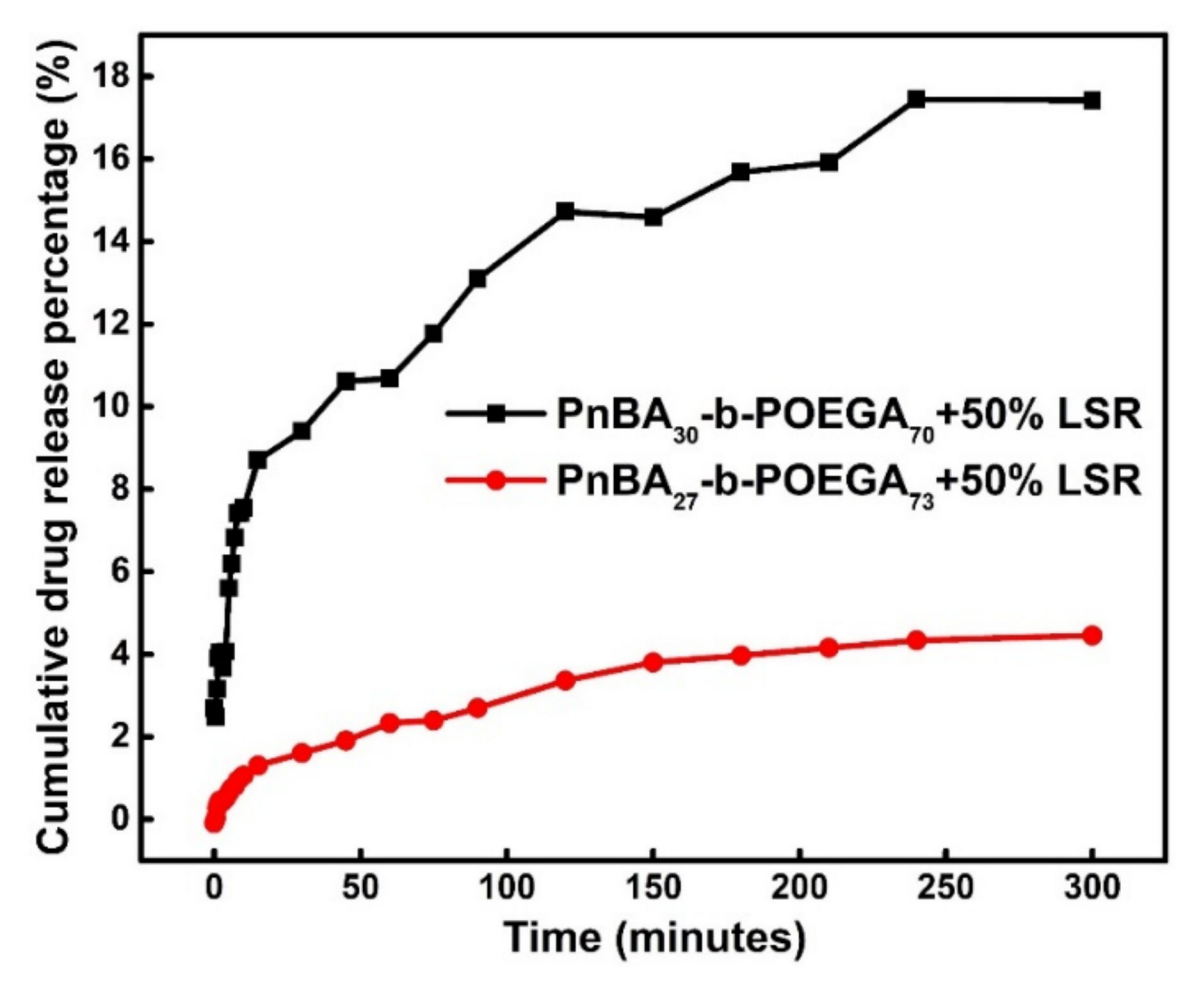
Release of LSR from PnBA_30_-b-POEGA_70_ and PnBA_27_-b-POEGA_73_ micelles in aqueous media under sonication.

The cumulative drug release percentage vs. time is provided in Figure 17 for the PnBA_30_-b-POEGA_70_ + 50% LSR (black line) and PnBA_27_-b-POEGA_73_ + 50% LSR (red line) nanocarriers. The release profile of LSR started simultaneously with the application of ultrasound, exhibiting a gradual increase in released LSR within five hours during the release experiments. Based on the UV-Vis results in Figure 17 for both nanocarriers, an initial burst release of LSR is observed in the first ten minutes, followed by a relatively gradual increase of released LSR until reaching a plateau. The relatively low LSR release (Table 7) implies that a large portion of the encapsulated LSR is tightly bound to the cores of polymeric PnBA_30_-b-POEGA_70_ and PnBA_27_-b-POEGA_73_ micelles, and it is quite difficult to release.

## 4. Conclusions

Novel biocompatible PnBA_30_-b-POEGA_70_ and PnBA_27_-b-POEGA_73_ AmBCs were successfully synthesized in different M_w_ of the PnBA block using RAFT polymerization, and highly stable drug-loaded nanocarriers were developed through TFHM. The PnBA-b-POEGA diblocks and biocompatible drug-loaded nanocarriers (formed by the encapsulation of LSR into amphiphilic PnBA-b-POEGA copolymers) were studied using a wide range of physicochemical methods.

The PnBA_30_-b-POEGA_70_ and PnBA_27_-b-POEGA_73_ diblock copolymers self-assembled into nano-sized core-shell micelles in aqueous milieu. The engulfing of LSR into the inner hydrophobic segments of copolymers contributed to the formation of greater size drug–polymer nanostructures.

Likewise, FBS interactions with PnBA-b-POEGA copolymers indicated a good stability of polymeric micelles in a biological environment, endorsing the idea that POEGA hydrophilic chains shield the copolymer micelles against protein adsorption and agglomeration. DLS stability studies for the LSR-loaded PnBA-b-POEGA nanocarriers suggested highly stable drug–polymer systems for a period of 23 days.

ATR-FTIR measurements verified the existence of LSR in the mixed copolymer–drug aqueous solutions by detecting new characteristic peaks of LSR in the ATR-FTIR spectra of the LSR-loaded PnBA-b-POEGA nanocarriers. Moreover, UV-Vis experiments also confirmed the successful encapsulation of LSR into the PnBA-b-POEGA micelles.

^1^H-NMR measurements further corroborated the presence of LSR into PnBA_30_-b-POEGA_70_ micelles in aqueous media. 2D-COSY, 2D-NOESY, and 2D-DOSY experiments confirmed the internal structure of the PnBA_30_-b-POEGA_70_ copolymer and LSR moieties and documented the successful encapsulation of LSR into the micelles. Specifically, 2D-NOESY experiments evidenced the strong intermolecular association between the biphenyl ring and butyl chain of LSR structure with the methylene signals of PnBA. Interestingly, the PnBA_30_-b-POEGA_70_ and PnBA_27_-b-POEGA_73_ micelles exhibited enhanced proton mobility, probably caused by partial loosening of the PnBA hydrophobic core upon temperature increase.

The highest hydrophobicity of the PnBA_27_-b-POEGA_73_ micelles contributed to an efficient encapsulation of LSR into the micelles exhibiting a greater value of %EE compared to PnBA_30_-b-POEGA_70_ + 50% LSR nanocarriers. Release rates of LSR implied that a significant amount of the encapsulated LSR is tightly bound to both PnBA_30_-b-POEGA_70_ and PnBA_27_-b-POEGA_73_ micelles, leading to relatively low release.

The current study presents an overall physicochemical characterization of novel block copolymer micellar carriers, including NMR measurements, which can accurately detect drug–copolymer intermolecular interactions in the system.

## Figures and Tables

**Table 1:** Molecular characteristics of PnBA homopolymers and PnBA-b-POEGA block copolymers.

Sample	M_w_^a^ (×10^3^ g/mL)	M_w_/M_n_^a^	M_w_^a^ (PnBA) (×10^3^ g/mL)	wt.% PnBA ^b^
PnBA-1	4.1	1.1	-	-
PnBA-2	7.8	1.27	-	-
PnBA_30_-b-POEGA_70_	12.900	1.29	4.1	30
PnBA_27_-b-POEGA_73_	28.600	1.47	7.8	27

^a^ Determined by SEC in THF/3%v Et_3_N. ^b^ Determined by ^1^H-NMR in CDCl_3._

**Table 2:** Results for PnBA_30_-b-POEGA_70_ and PnBA_27_-b-POEGA_73_ micelles in aqueous solutions as obtained by DLS (dynamic light scattering), SLS (static light scattering), ELS (electrophoretic light scattering), and FS (fluorescence spectroscopy) techniques.

Sample	R_h_^a^ (nm)	PDI ^a^	Intensity ^a^ (a.u)	N_agg_^b^	CMC ^c^ (g/mL)	ζ_pot_^d^ (mV)	L ^e^ (nm)
PnBA_30_-b-POEGA_70_	10	0.12	167	25	1.09 × 10^−5^	−1	12
PnBA_27_-b-POEGA_73_	24	0.15	960	133	1.26 × 10^−6^	−7	25

^a^ Determined by DLS at measuring angle 90°, ^b^ Determined by SLS, ^c^ Determined by FS, ^d^ Determined by ELS, ^e^ Calculated length of the fully extended macromolecular chain based on the total number of monomers multiplied by 0.254 nm.

**Table 3:** Physicochemical characteristics of FBS and protein–polymer mixtures obtained from DLS measurements based on the protocol used for their preparation. Data for R_h_ denote multiple peaks.

Sample	Protocol	R_h_ (nm)	PDI	Intensity (a.u)
FBS	-	4 23 170	0.50	5760
PnBA_30_-b-POEGA_70_ + FBS:PBS (1:9 *v*/*v*)	1	3 10 96	0.46	1032
PnBA_27_-b-POEGA_73_ + FBS:PBS (1:9 *v*/*v*)	1	8 60	0.47	1061
PnBA_30_-b-POEGA_70_ + FBS:PBS (1:9 *v*/*v*)	2	3 10 80	0.49	1019
PnBA_27_-b-POEGA_73_ + FBS:PBS (1:9 *v*/*v*)	2	4 17	0.47	964
PnBA_30_-b-POEGA_70_ + FBS:PBS (1:1 *v*/*v*)	2	4 17 120	0.49	3740
PnBA_27_-b-POEGA_73_ + FBS:PBS (1:1 *v*/*v*)	2	18	0.48	3880

**Table 4:** Results for the LSR-loaded PnBA-b-POEGA nanocarriers in aqueous solutions.

Sample	R_h_^a^ (nm)	PDI ^a^	Intensity ^a^ (a.u)	ζ_pot_^b^ (mV)
PnBA_30_-b-POEGA_70_ + 20%LSR	12	0.36	116	−13
PnBA_30_-b-POEGA_70_ + 50%LSR	30	0.30	215	−23
PnBA_27_-b-POEGA_73_ + 20%LSR	28	0.19	471	−14
PnBA_27_-b-POEGA_73_ + 50%LSR	22	0.22	396	−16

^a^ Determined by DLS at measuring angle 90°, ^b^ determined by ELS.

**Table 5:** ^1^H-NMR chemical shifts of PnBA_30_-b-POEGA_70_ copolymer: (**a**) in the absence of LSR and (**b**) in the presence of LSR in D_2_O solutions.

		^1^H-NMR Chemical Shifts (ppm)
Protons of the Copolymer Structure	(a) Proton Signals of PnBA_30_-b-POEGA_70_ in the Absence of LSR	(b) Proton Signals of PnBA_30_-b-POEGA_70_ in the Presence of LSR
Ha–b	2.13	2.07
Hc	3.94	3.89
Hd	1.51	1.46
He	1.34	1.28
Hf	0.86	0.81
Hg	4.17	4.11
Hh	3.60	3.54
Hi	3.28	3.22
*Reference*	D_2_O: 4.69	

**Table 6:** ^1^H-NMR chemical shifts of LSR: (**a**) in the presence of PnBA_30_-b-POEGA_70_ copolymer and (**b**) in the presence of SDS (sodium dodecyl sulfate) micelles in D_2_O solutions.

		^1^H-NMR Chemical Shifts (ppm)
Protons of LSR Structure	(a) Proton Signals of LSR in PnBA_30_-b-POEGA_70_	(b) Proton Signals of LSR in SDS Micelles
H1	0.63	0.77
H2	1.13	1.22
H3	1.32	1.5
H4	2.43	2.52
H5	4.35	4.39
H7	5.11	5.15
H8−H9	6.78	6.85
H10–H11	6.93	6.99
H12–H13	7.45	7.64
H14–H15	7.38	7.34–7.41
*Reference*	D_2_O: 4.69	

**Table 7:** Drug encapsulation and release characteristics of LSR loaded in PnBA_30_-b-POEGA_70_ and PnBA_27_-b-POEGA_73_ micelles in water.

Sample	%DL	%EE	%Maximum Release Rate of LSR
PnBA_30_-b-POEGA_70_ + 50%LSR	1	6	17
PnBA_27_-b-POEGA_73_ + 50%LSR	4	25	5

## Data Availability

The data presented in this study are available on request from the corresponding author.

## Supplementary Materials

The following are available online at https://www.mdpi.com/article/10.3390/polym13071164/s1, Figure S1: comparative diagram of the calculated relative intensity ratio I1/I3 of pyrene peaks versus the copolymer concentration in water for the PnBA30-b-POEGA70 and PnBA27-b-POEGA73 copolymers; Figure S2: (a) comparative size distributions of bare PnBA27-b-POEGA73 micelles and FBS, (b) intensity size distributions of PnBA27-b-POEGA73 + FBS:PBS (1/9 v/v) using protocol 1, (c) size distributions of PnBA27-b-POEGA73 + FBS:PBS (1/9 v/v) using protocol 2, and (d) size distributions of PnBA27-b -POEGA73 + FBS:PBS (1/1 v/v) using protocol 2; Figure S3: Rh and intensity measurements as a function of temperature for the PnBA30-b-POEGA70 block copolymers in aqueous solutions at 90 degrees; Figure S4: 1H-NMR chemical shifts as a function of temperature for the PnBA_30_-b-POEGA_70_ block co-polymers
