# Supplementary material for: Nano-Assemblies from Amphiphilic PnBA-b-POEGA Copolymers as Drug Nanocarriers
Source: Polymers (Basel). 2021 Apr 5;13(7):1164. doi: 10.3390/polym13071164 (PMC8038588; doi:10.3390/polym13071164)
Supplement: Supplementary file 1 [file polymers-13-01164-s001.pdf]

polymers

MDPI

Supplementary Material: Nano-Assemblies from Amphiphilic PnBA-b-POEGA Copolymers as Drug Nanocarriers

Angeliki Chroni, Thomas Mavromoustakos and Stergios Pispas

1. Results

1.1. Physicochemical Characterization of the PnBA-b-POEGA Micelles

With the purpose of examining the versatile properties of the PnBA-b-POEGA block copolymers, critical micelle concentration (CMC) values were determined using the FS technique with pyrene as the fluorescent probe. The polymer stock solutions were prepared at 10^{-3} g/ml and pH = 7. The solution concentrations were prepared in a range of 1 x 10^{-8} to 1 x 10^{-3} g/ml. The calculated relative intensity ratio I_1/I_3 of Py peaks versus the copolymer concentration in water for the $PnBA_{30}$-b-$POEGA_{70}$ and $PnBA_{27}$-b-$POEGA_{73}$ copolymers is shown in Figure S1. Higher M_w of PnBA (7800) induces a significant reduction in the CMC value of $PnBA_{27}$-b-$POEGA_{73}$ diblock (red line) compared to $PnBA_{30}$-b-$POEGA_{70}$ diblock (black line), which is evident in Figure S1 and consistent with the literature data [1]. Both CMC values are presented in the main text in Table 2.

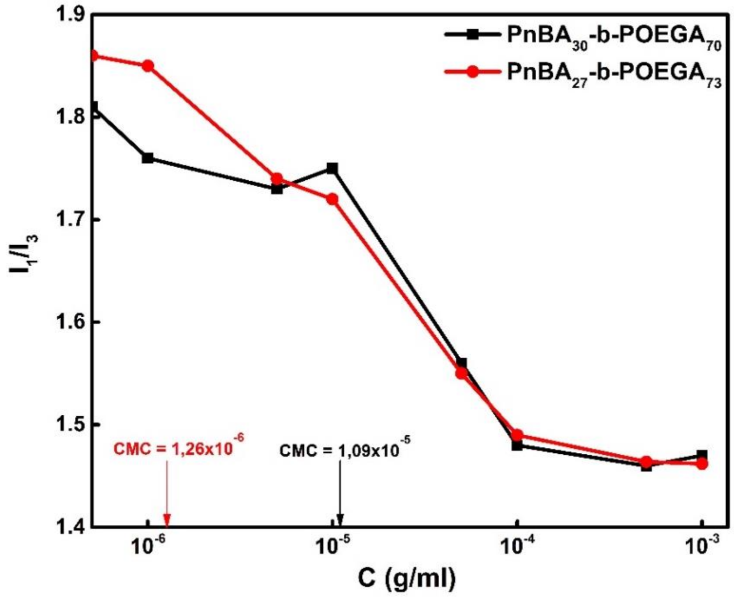

Figure S1. Comparative diagram of calculated relative intensity ratio I_1/I_3 of pyrene peaks versus the copolymer concentration in water for the $PnBA_{30}$-b-$POEGA_{70}$ and $PnBA_{27}$-b-$POEGA_{73}$ copolymers.

1.2. FBS Interactions with PnBA-b-POEGA Block Copolymers

DLS stability studies of $PnBA_{27}$-b-$POEGA_{73}$ micelles in FBS solutions were performed to assess the R_h, PDI, and Intensity values of the protein–polymer mixtures. A comparison of intensity size distributions of bare $PnBA_{27}$-b-$POEGA_{73}$ (red line) micelles and FBS (black line) is exhibited in Figure S2a. The analysis of trimodal size distributions of FBS is reported in the main text. Protocol 1 [mixing of 50 μl sample with 3 mL FBS:PBS (1/9 v/v)] used for the preparation of protein–polymer mixtures is denoted in Figure S2b, whereas protocol 2 [mixing of 100 μl sample with 3 mL FBS:PBS (1/9 v/v) and 3 mL FBS:PBS (1/1 v/v)] in is denoted in Figures S2c to S2d. Only a few conclusions can be drawn after the incubation of $PnBA_{27}$-b-$POEGA_{73}$ micelles with FBS (for both protocols)

due to the intrinsic difficulty in separating the DLS signals of the bare micelles (24 nm) and the second population of FBS (23 nm). A bimodal distribution appeared in Figure S2b, corresponding to protocol 1, with peaks emerging at 8 nm (possibly free BSA) and 60 nm (possibly protein–polymer complexes). Based on protocol 2 at FBS:PBS (1/9 v/v) ratio, a co-existence of free serum proteins at 4 nm and decreased size micelles at 17 nm is depicted in Figure S2c. A rather broad size distribution emerged in Figure S2d at FBS:PBS (1/1 v/v) ratio using protocol 2, the size of which is slightly decreased (18 nm) compared to bare micelles (24 nm). Ultimately, the $PnBA_{27}$-b-$POEGA_{73}$ micelles maintained their synthetic identity after the incubation with FBS, indicating good stability in a biological medium.

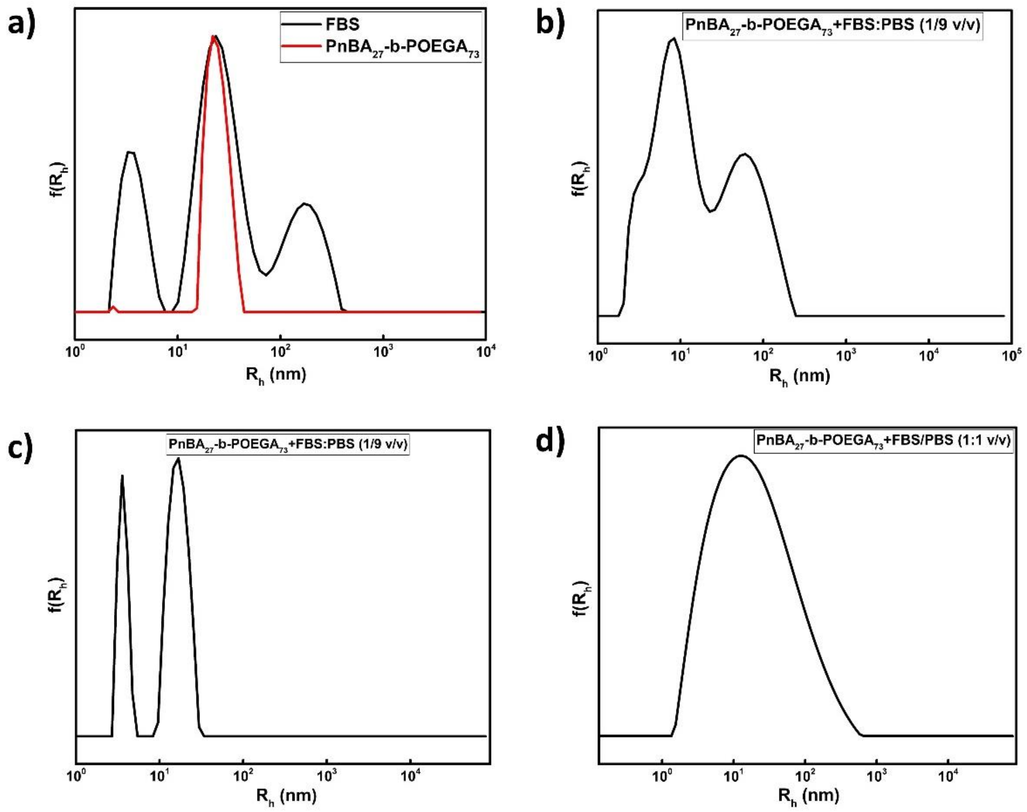

1.3. DLS measurements as a Function of Temperature

Figure S2. a) Comparative size distributions of bare $PnBA_{27}$-b-$POEGA_{73}$ micelles and FBS, b) intensity size distributions of $PnBA_{27}$-b-$POEGA_{73}$+FBS:PBS (1/9 v/v) using protocol 1, c) size distributions of $PnBA_{27}$-b-$POEGA_{73}$+FBS:PBS (1/9 v/v) using protocol 2, and d) size distributions of $PnBA_{27}$-b -$POEGA_{73}$+FBS:PBS (1/1 v/v) using protocol 2.

Aqueous micellar solutions of PnBA-b-POEGA block copolymers were prepared and characterized using DLS to further probe the effect of temperature on their self-assembly. The measurements were performed at a concentration of 10^{-3} g / ml and pH = 7 and at a temperature range of 25 °C–55 °C. Figure S3 exhibits the R_h and intensity measurements as a function of temperature at a 90° angle for the $PnBA_{30}$-b-$POEGA_{70}$ diblock copolymer. The scattered light intensity slightly increases as temperature rises from 25 °C to 55 °C, resulting to the formation of increased-mass nanostructures. In parallel, the R_h decreases with increasing temperature due to the shrinkage of polymer chains and the intrinsic aggregation tendency of the system. In relation to DLS data, the $PnBA_{30}$-b-$POEGA_{70}$ micelles may be considered temperature-independent.

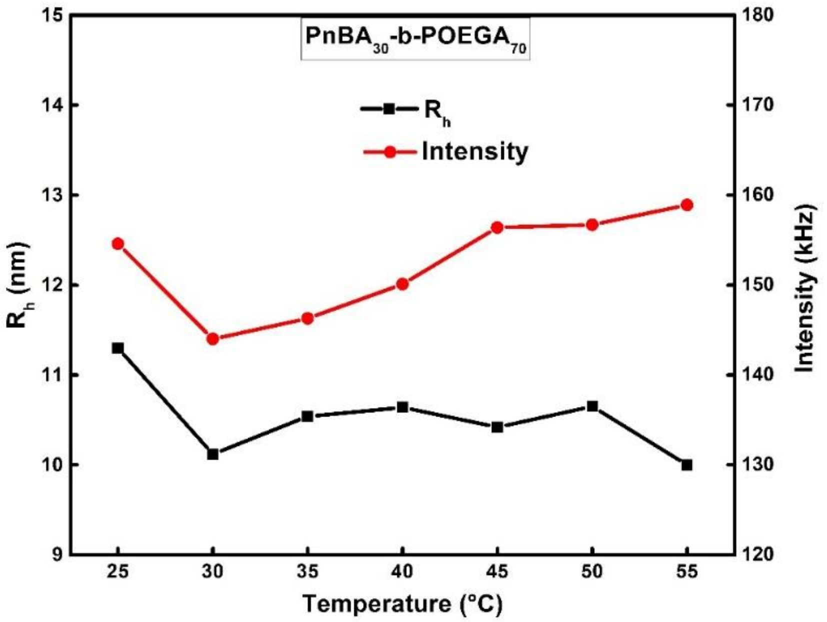

Figure S3. R_h and Intensity measurements as a function of temperature for the $PnBA_{30}$-b-$POEGA_{70}$ block copolymers in aqueous solutions at 90 degrees.

1.4. Chemical Shift as a Function of Temperature

The changes in chemical shift as a function of the temperature of $PnBA_{30}$-b-$POEGA_{70}$ block copolymers is presented in Figure S4 to provide a clearer view of the shift of the ^{1}H-NMR spectra (Figure 15 exhibited in the main text) to the lower field regions.

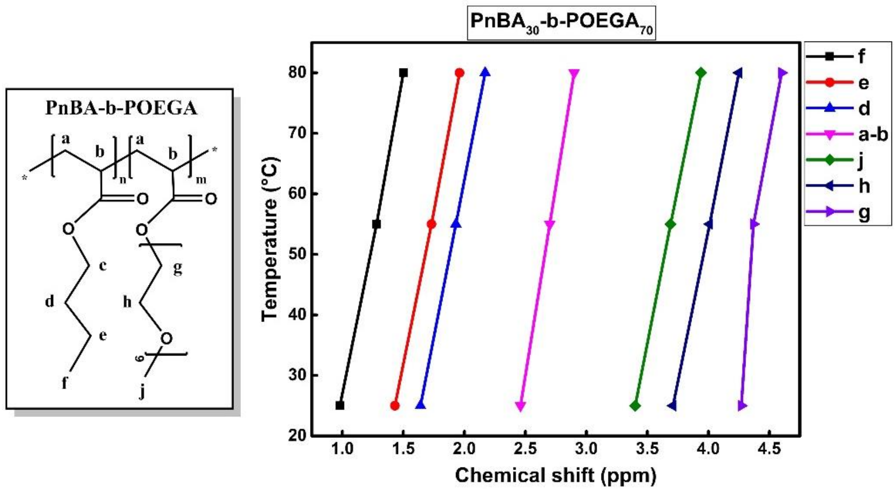

Figure S4. ^{1}H-NMR chemical shifts as a function of temperature for the $PnBA_{30}$-b-$POEGA_{70}$ block copolymers.

References

1. Antoun, S.; Gohy, J.-F.; Jérôme, R. Micellization of quaternized poly (2-(dimethylamino) ethyl methacrylate)-block-poly (methyl methacrylate) copolymers in water. *Polymer* **2001**, *42*, 3641-3648, doi:10.1016/S0032-3861(00)00746-1.
